# Improving Inference Within Freshwater Community Studies: Accounting for Variable Detection Rates of Amphibians and Fish

**DOI:** 10.1002/ece3.70383

**Published:** 2024-10-16

**Authors:** Andrew J. Hamer, Júlia Horányi

**Affiliations:** ^1^ Institute of Aquatic Ecology, HUN‐REN Centre for Ecological Research Budapest Hungary; ^2^ National Multidisciplinary Laboratory for Climate Change, HUN‐REN Centre for Ecological Research Budapest Hungary

**Keywords:** aquatic habitat, occupancy probability, phenology, rare species, survey methodology, urbanisation

## Abstract

Research into freshwater communities often aims to link patterns of species distribution in ponds with underlying biotic factors. However, errors with species detection (e.g. false negatives) may underestimate distribution and bias assessments of community structure. Occupancy models that account for imperfect detection offer a solution to this problem. Here, we used three methods (call/visual encounter surveys, dip‐netting and newt trapping) to survey amphibians and fish (potential amphibian predators) at 100 ponds in an urbanised landscape in Hungary over one breeding season. We estimated species detection probabilities for amphibians (all life stages combined) and fish using occupancy models to gain insight into amphibian‐fish relationships and other survey‐specific variables. We detected nine amphibian and 20 fish species. There were relatively low but variable estimated probabilities of detection for amphibians (mean: 0.320, 95% Bayesian credible interval: 0.142–0.598), with three species having detection rates < 0.1. Probabilities of detection peaked in the middle of the breeding season and increased with survey effort. Detection probabilities of five species were negatively associated with the detection of fish at a pond, while there were positive relationships between detection and emergent vegetation cover. We found no substantial differences in detection rates among the three survey methods. The probability of detecting fish was much higher than for amphibians (0.588, 0.503–0.717) but was lower at ponds with high emergent vegetation where amphibian detection was higher. Our results underscore the importance of accounting for the imperfect detection of both response organisms and potentially interacting species in aquatic community studies. We recommend applying multi‐species occupancy models to enable inference for both common and rare species at ponds in landscapes subjected to human disturbances.

## Graphical abstract

We used three sampling methods at freshwater ponds to survey amphibian and fish communities in an urbanised landscape. We found highly variable detection probabilities among nine amphibian species and higher detection rates for fish. The detection of fish at a pond reduced the probability of detecting amphibians. However, emergent vegetation decreased the chance of detecting fish while increasing the probability of detecting amphibians. Our results underscore the importance of accounting for imperfect detection when assessing amphibian‐fish relationships.

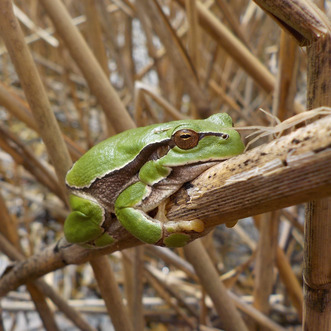

## 1 Introduction

Accounting for imperfect detection is crucial for robust inference regarding the occurrence of species in pond communities. One of the criticisms levelled at the elements of metacommunity structure (EMS), which seeks to identify structural patterns in community datasets (Leibold and Mikkelson 2002), was that problems with species detection could lead to inaccurate assessments of metacommunity structure (Mihaljevic, Joseph, and Johnson 2015). For instance, failing to detect a species during a survey at a site when it is actually present can result in false negatives that underestimate occupancy. However, modelling of species occurrence at a site over repeated sampling occasions enables an estimation of true occupancy corrected for the probability of a species being detected at the site, which forms the basis of occupancy models (MacKenzie et al. 2002). Furthermore, multi‐species occupancy modelling improves understanding of metacommunity structure by allowing estimation of species‐specific covariate effects on the probabilities of occupancy (Dorazio et al. 2010; Mihaljevic, Joseph, and Johnson 2015). Estimating the probability of species detection in studies of community composition is particularly important when dealing with rare or infrequently observed species that either have low population sizes or patchy distribution patterns (MacKenzie et al. 2005; Thompson 2004).

Amphibians represent one of the most imperilled vertebrate groups (Stuart et al. 2004), and many species have declining populations resulting from the variable effects of environmental stressors at regional scales (Grant et al. 2016; Houlahan et al. 2000). Owing to their biphasic life cycle, with aquatic larvae and largely terrestrial habits post‐metamorphosis, many pond‐breeding amphibians are vulnerable to habitat loss and fragmentation between ponds and uplands (Cushman 2006), with communities at particular risk being in urbanising landscapes (Hamer and McDonnell 2008). Many species have low probabilities of detection in modified landscapes (e.g. Guzy et al. 2019; Lima et al. 2019; Ribeiro Jr. et al. 2018; Sankararaman et al. 2021). Therefore, studies of amphibian communities need to account for imperfect detection of species during field surveys.

A range of factors cause heterogeneity in species detection rates, including individual characteristics (e.g. rarity, behaviour), spatial variation (e.g. habitat quality), temporal variation (e.g. seasonality, weather, time of day) or survey methodology and effort (Guillera‐Arroita 2017). Amphibian studies commonly consider the effects of seasonality (i.e. breeding phenology) and weather conditions (e.g. temperature, rain) on detection probabilities, with a moderate number including habitat variables, and a smaller number assessing the effects of survey methodology and effort (Schmidt et al. 2023). For example, studies have evaluated detection probabilities among different survey techniques for amphibians (Bailey, Simons, and Pollock 2004; Baumgardt et al. 2021; Sewell, Beebee, and Griffiths 2010), while others have shown that small population sizes can reduce detection rates (Tanadini and Schmidt 2011).

Variable population sizes induce heterogeneity in site‐specific detection probabilities, and species that are more abundant within a site are generally easier to detect (Royle and Nichols 2003). Many amphibian species are vulnerable to predation by fish (Falaschi et al. 2020) that can decrease abundance and local population sizes (Hamer and Parris 2013; Schmidt et al. 2021), sometimes to extinction (Knapp and Matthews 2000). Therefore, the presence of predatory fish species is often an important determinant of amphibian community structure and turnover in freshwater ponds (Snodgrass, Bryan, and Burger 2000; Wellborn, Skelly, and Werner 1996; Werner et al. 2007). Given that predation by fish on amphibians can reduce the abundance of some species, the presence of fish could reduce amphibian detectability. Few studies, however, have included the detection of a predator in estimating amphibian detection probabilities, despite including fish presence when modelling occupancy (but see Holgerson et al. 2019; Rowe et al. 2019). Furthermore, we are aware of only two studies that modelled fish detection and occupancy separately from amphibians (Amburgey et al. 2014; Rowe et al. 2019). One study found that non‐native fish were detected at 24% of sites, prior to correcting for imperfect detection, whereas the modelled probability of fish occupancy at sites with moderate water depth (1–2 m) was much higher at 85% (Rowe et al. 2019). This result highlights the importance of considering fish detection because sampling methods commonly used for amphibians may underestimate fish occupancy.

Here, we used three survey methods (call/visual encounter surveys, dip‐netting and newt trapping) to sample amphibian communities on repeated occasions across a highly urbanised landscape to examine relationships between detection probabilities and survey‐specific covariates representing variation in seasonal, climatic and habitat factors. We assessed differences in detection probabilities among survey methods to determine the efficacy of each technique. We also estimated the detection probability of fish and evaluated relationships between the detection of fish and survey‐specific covariates, as well as the relationship between the detection of amphibians and fish. Given the landscape context, we predicted that detection probabilities would be highly variable across the amphibian community due to species rarity and/or low abundance. We also predicted that fish would reduce the probability of amphibian detection at ponds. We consider the implications of our results for the design of occupancy studies, especially when allocating survey efforts to detect rare species. We also discuss the implications for studies assessing the impacts of fish on amphibian occupancy at ponds.

## 2 Methods

### 2.1 Study Area

**FIGURE 1:**
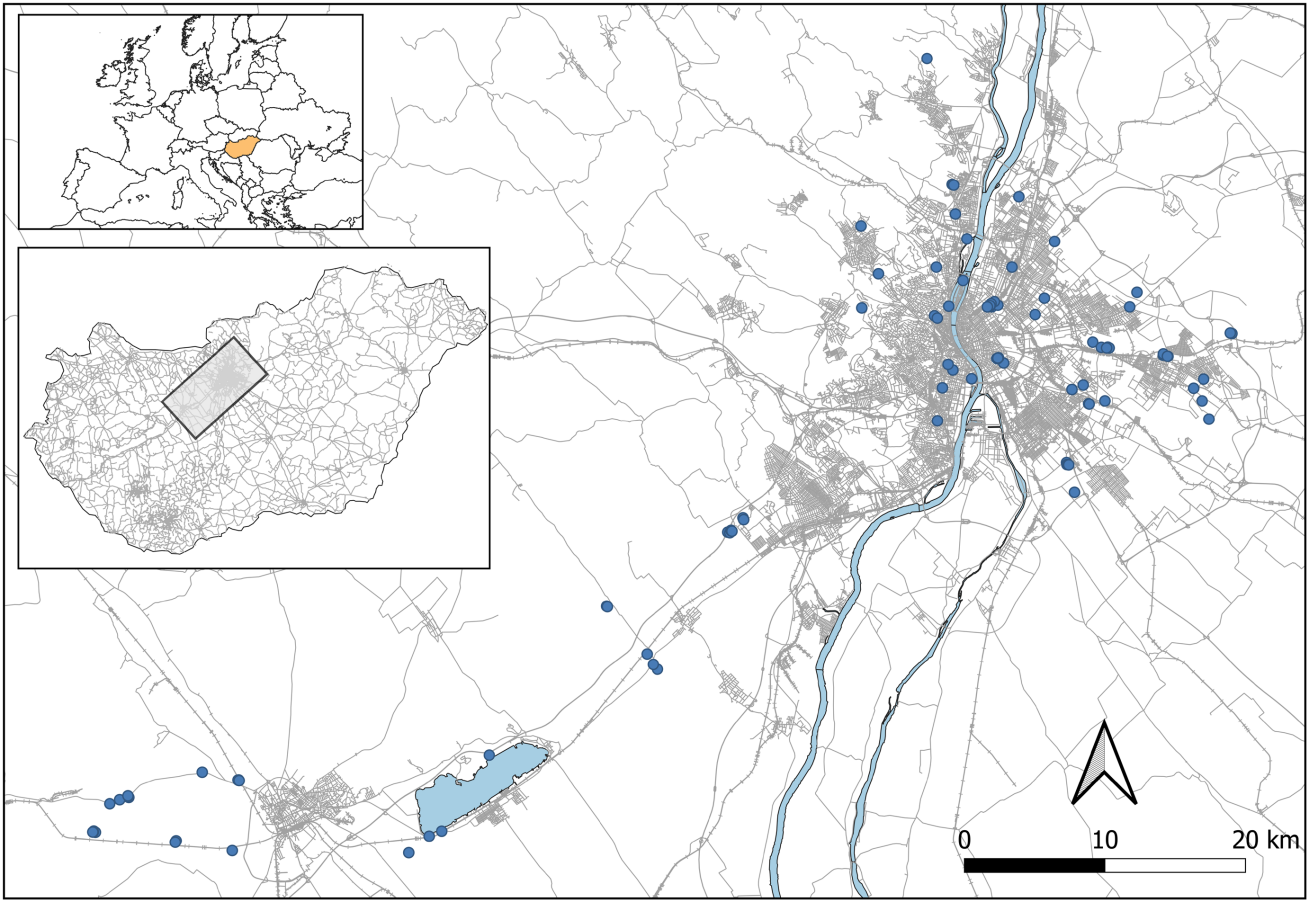
Map of the study area showing the locations of the 100 ponds in central Hungary (Budapest). The road and rail network is shown in grey.

The study area extended around Budapest (the capital city of Hungary) and 70 km to the south‐west (Figure 1). The study area was composed of urban, agricultural and forested land predominantly situated in low‐lying areas (< 450 m above sea level) within the Pannonian Biogeographical Region. The climate is moderately warm and dry, with an annual mean temperature of ~11°C and mean annual precipitation of 533 mm (Tóth‐Ronkay et al. 2015). The Danube River intersects the study area through Budapest flowing from north to south, while Lake Velence occurs in the south‐west (Figure 1). Wetland destruction in Hungary has been widespread since the nineteenth century (97% loss of original wetlands), initially due to river regulation leading to wetland drying, and more recently due to large‐scale urbanisation and road construction, with negative impacts on the 17 recorded amphibian species (Tóth‐Ronkay et al. 2015; Vörös, Kiss, and Puky 2015). The introduction of fish that prey on the larvae and adults of native amphibians also threatens species (Vörös, Kiss, and Puky 2015).

### 2.2 Site Selection

A total of 100 waterbody sites (herein ‘ponds’) were selected including 30 ponds in the south‐west portion of the study area previously surveyed by one of the authors in 2020 (Hamer, Barta, et al. 2021), and 55 ponds surveyed around Budapest in 2022 as part of a citizen science monitoring project (www.mypond.hu), plus an additional 15 ponds (Figure 1). Ponds were originally selected using Google Earth Pro images (Google Inc. 2023) and the Ecosystem Base Map of Hungary (Ministry of Agriculture 2019), with the final set of sites ultimately determined by property access. Site selection ensured there was a wide range in pond surface area (mean: 1843 m^2^, range: 6–20,614 m^2^) and the proportion cover of urban land within a 1‐km radius around ponds (mean: 0.372, range: 0.001–0.898), with measurements calculated using QGIS v3.30.3 (QGIS Development Team 2022). Hence, the ponds were distributed over a broad geographical region and along multiple urban–rural gradients. A variety of pond types was sampled (e.g. ornamental ponds, fish ponds, stormwater retention ponds, agricultural ponds, floodplain ponds) including natural/semi‐natural and artificial, as well as permanent and ephemeral ponds. Ponds were grouped into 20 spatial clusters comprised of 3–8 sites, based on the distance to the nearest neighbouring site (mean distance: 807 m, range: 3–9058 m).

### 2.3 Amphibian Surveys

Three surveys were conducted at the 100 sites over a single breeding season in spring and summer 2023 (survey 1: March—April; survey 2: May—June; survey 3: July—August), corresponding with the breeding season of amphibian species recorded in the region (Berninghausen and Berninghausen 2001). The three surveys were conducted at a large number of sites to increase the probability of detecting both common and rare species (MacKenzie and Royle 2005). Ponds within the same spatial cluster were generally surveyed on the same date, and the order in which clusters were surveyed was randomised. We used three survey methods to detect all life stages: (1) call/visual encounter surveys (VES), (2) dip‐netting, and (3) Dewsbury newt traps. Surveys were conducted during the daytime (between 0800 and 1700 h). We started our surveys with a 5‐min call survey quietly listening for anuran calls while standing near the pond shoreline. Prior to or during dip‐netting, we conducted slow‐paced visual searches (VES) of the water column, emergent vegetation and shoreline vegetation within 10 m of the pond bank for eggs, larvae and metamorphosed, juvenile and adult amphibians (Crump and Scott Jr. 1994). Next, dip‐netting began at waterbodies with sufficient water levels (water depth > 10 cm) using a net design appropriate for the safe capture of amphibians and their larvae (300‐mm wide frame, 350 mm deep, 2 mm mesh size). The number of net sweeps was scaled to the pond surface area—one dip‐net sweep per 25 m^2^ of water surface area (Shulse et al. 2010), with a minimum of two sweeps and a maximum of 213 sweeps. The number of sweeps at a site was reduced if declining water levels had decreased the water surface area. Each dip‐net sweep was approximately 1.5 m in length, performed in all available microhabitat types at various water depths (e.g. open water, patches of emergent vegetation) to target the aquatic microhabitat preferences of amphibians (Shaffer et al. 1994). Dip‐net sweeps occurred over the entire surface area of shallow ponds (< 1.2 m deep) but were restricted to the shoreline in deeper ponds.

Dewsbury traps were deployed at the bottom of ponds > 300 m^2^ in area and > 20 cm deep to capture newts but also other amphibians (Dewsbury 2014). The traps consisted of a small plastic box (25 × 17 × 12 cm) with a square funnel entrance made of plastic mesh. The box was attached to a 76 cm‐long plastic bag containing a float that hung vertically in the water column to provide a breathing space for trapped individuals. The number of newt traps deployed was proportional to pond area (2 traps minimum, plus 1–2 additional traps for increases in area greater than 3000 m^2^; modified sampling protocol from Adams, Richter, and Leonard 1997). Newt traps were not deployed in public ponds where there was a risk of theft or interference. Traps were deployed during or following dip‐netting and retrieved the next morning. Traps were positioned in optimal microhabitats for capturing newts (e.g. patches of emergent or submerged vegetation), but only at the shoreline in deep ponds (> 1.2 m deep). Start and stop times for each survey method were recorded. Dip‐netting and trapping were not conducted at dry ponds, although VES were done.

We identified and counted amphibian species (including egg masses), and then released individuals unmarked at the point of capture. No voucher specimens were taken for preservation. In some smaller waterbodies, amphibian larvae caught in dip‐nets were held temporarily in a plastic bucket and then identified, counted and released. We recorded fish species captured in dip‐nets and newt traps or seen in the water column. While dip‐netting and trapping might not detect larger‐bodied fish, visual searches of open water were considered sufficient for detecting larger fish species. Amphibians were identified to species using Berninghausen and Berninghausen (2001). Taxonomy follows Speybroeck et al. (2020). Green frogs (*Pelophylax lessonae*, *P. esculentus* and *P. ridibundus*) were consolidated within the *Pelophylax* spp. complex as the species cannot be distinguished easily in the field, especially larvae. Fish species were identified using Harka and Sallai (2004). Standard hygiene protocols to minimise the risk of spreading the amphibian chytrid fungus (*Batrachochytrium dendrobatidis* and *B. salamandrivorans*) were followed when conducting fieldwork, with all equipment disinfected with Virkon solution (Phillott et al. 2010).

### 2.4 Survey Covariates

Water temperature was recorded at the start of call surveys, 1 m from the shoreline at a depth of ~10 cm. Water levels were recorded during each survey as a percentage of the full water‐holding capacity of a site, with the shoreline delineated by bank morphology, visible high‐water line or vegetation type. Wind speed was recorded in the field using a modified Beaufort scale (0–3) but was reduced to a binary variable for modelling (see Halstead, Rose, and Kleeman 2021). Rainfall was recorded during a survey as a binary variable. These survey parameters were measured because amphibian detectability is often affected by rain, temperature and wind (Weir et al. 2005), and the availability of water at a breeding site (Alford and Richards 1999). The study was not conducted during a drought period. We recorded survey‐specific detections of fish but did not include larvae (fry) in the analyses, given that they pose no threat to amphibians (Kloskowski 2010). We visually estimated the percentage of the pond surface area covered by emergent vegetation each survey, including any vegetation (e.g. reeds, sedges) that extended above the water surface. Studies of amphibian communities in ponds have found positive and negative effects of both fish and emergent vegetation on species' detection probabilities (Curtis and Paton 2010; Holgerson et al. 2019).

### 2.5 Modelling of Detection Probabilities

Multi‐species occupancy models (MSOM) with Bayesian inference were used to estimate the probabilities of detection at the community and individual species levels. Hierarchical MSOM estimate community‐level parameters by estimating species‐specific probabilities of detection (Dorazio and Royle 2005; Dorazio et al. 2006), and address the issue of imperfect species detection in metacommunity studies (Mihaljevic, Joseph, and Johnson 2015). Detection models for individual species are linked together into an overarching model so that collectively they provide estimates of community‐level responses to survey and site covariates, which increases the precision of parameter estimates for infrequently observed species (Dorazio et al. 2006; Kéry and Royle 2008; Zipkin, DeWan, and Andrew Royle 2009). Data from species frequently detected at sites is shared across the community to improve the predictive ability of parameter estimates for rare species (Zipkin, DeWan, and Andrew Royle 2009), which results in the mean estimates of data‐poor species being drawn towards group averages (Link 1999).

Multi‐species occupancy models were used to examine relationships between the probabilities of detection of any life stage (i.e. eggs, larvae, juveniles or adults) and nine covariates recorded during a survey: (1) the number of days since 19 March 2023 (Days); (2) a quadratic relationship of the number of days (Days^2^); (3) survey effort (Effort); (4) water temperature (Temp); (5) water levels (Water); (6) wind speed (Wind); (7) rain (Rain); (8) detection/non‐detection of fish (Fish); and (9) emergent vegetation cover (Emergent). The first survey occurred on 20 March (i.e. day 1), with the number of days calculated from 19 March (i.e. day 0) to each survey date. This covariate was included both as a linear and quadratic term to account for the seasonal patterns in detectability among species (Curtis and Paton 2010; Petitot et al. 2014). Inclusion of number of days also accounted for differences in species' availability over the breeding season, thereby addressing the closure assumption in occupancy modelling (see Schmidt 2005; Petitot et al. 2014). The first MSOM was built using detection data from all three survey methods and the nine covariates (Model 1), examining relationships between detection probabilities and the full set of survey covariates. An additional three separate models were built to assess differences in the probabilities of detection among the three survey methods, and included three covariates that had the greatest influence on detection (Model 2: VES; Model 3: Dip‐netting; Model 4: Newt trapping). Survey effort in Models 1 and 2 was expressed as the number of minutes spent conducting VES, which were always done at a site even if dry, and was moderately to strongly correlated with the number of dip‐net sweeps and number of traps at a site (*r* ≥ 0.5; Table S1). Survey effort in Models 3 and 4 was included as the number of dip‐net sweeps and number of traps for each survey, respectively. Species were excluded from Models 2–4 if they were not detected using that particular survey method. Detection probabilities for each method were not compared in a single MSOM because detection data from the three survey techniques were not independent (Bailey, Simons, and Pollock 2004; Otto and Roloff 2011). However, Guzy, Price, and Dorcas (2014) assessed detection probabilities using a model developed for multiple detection methods and found strong evidence of variation in anuran detection probabilities between call surveys and visual encounter surveys. Accordingly, we combined call and visual surveys under the VES method.

The first level of hierarchy of each MSOM was based on an occupancy sub‐model that assumed a true (but only partially observed) presence–absence matrix *z*_*i,j*_ for species *i* = 1, 2,…, *N* at site *j* = 1, 2,…, *J*, where *z*_*i,j*_ = 1 if species *i* was present at site *j*, and *z*_*i,j*_ = 0 if the species was absent (Dorazio et al. 2006; Kéry and Royle 2008). Because *z*_*i,j*_ was uncertain, a species‐specific occurrence model was specified where *z*_*i,j*_ ~ Bernoulli(Ψ_*i,j*_), and Ψ_*i,j*_ is the probability that species *i* occurs at site *j*. The state variable *z*_*i,j*_ is usually not known with certainty; instead, observed data *x*_*i,j,k*_ was used for species *i* at site *j* during sampling period *k*, which are also assumed to be Bernoulli random variables if species *i* is present (Dorazio et al. 2006). Thus, the data provided a three dimensional matrix *x*_*i,j,k*_ for species *i* at site *j* for the *k*th sampling occasion. The second level of model hierarchy was based on an observation (detection) sub‐model, which specified that *x*_*i,j,k*_ ~ Bernoulli(*p*_*i,j,k*_ × *z*_*i,j*_) where *z*_*i,j*_ is the true occurrence matrix described above, and *p*_*i,j,k*_ is the detection probability for species *i* at site *j* for the *k*th sampling occasion (Dorazio et al. 2006).

The occupancy sub‐model included only an intercept term:

$$\text{logit}\left(\Psi_{i,j}\right)=\alpha_{0i}$$

In Model 1, detection probability was modelled as a linear function of the nine survey‐specific covariates:

$$\begin{array}{cc} \text{logit}\left(p_{i,j,k}\right) & \;=\beta_{0i}+\beta_{1i}\;\left({\text{Days}}_{j,k}\right)+\beta_{2i}\;\left({{\text{Days}}^{2}}_{j,k}\right)+\beta_{3i}\;\left({\text{Effort}}_{j,k}\right)\\ & +\beta_{4i}\;\left({\text{Temp}}_{j,k}\right)+\beta_{5i}\;\left({\text{Water}}_{j,k}\right)+\beta_{6i}\;\left({\text{Wind}}_{j,k}\right)\\ & +\beta_{7i}\;\left({\text{Rain}}_{j,k}\right)+\beta_{8i}\;\left({\text{Fish}}_{j,k}\right)+\beta_{9i}\;\left({\text{Emergent}}_{j,k}\right)+\varepsilon_{i,j,k} \end{array}$$

where the coefficients *α*_0*i*_ and *β*_0*i*_ are species‐level effects (intercept terms) on the probability of occupancy and detection (respectively); *β*_1*i…*9*i*_ are survey‐level effects of the nine detection covariates, and *ε*_*i,j,k*_ is a term for random effects in detectability across species, sites and surveys (Kéry et al. 2009). Models 2–4 included the intercept terms and random effects but only *β*_1*i…*3*i*_ as survey‐level effects of three detection covariates. Lastly, a single‐species occupancy model (MacKenzie et al. 2002) was used to assess relationships between the probability of detecting fish from all three survey methods (Model 5: all species combined, except fry) and eight survey covariates included in Model 1 (all except Fish covariate). As done in Models 1–4, occupancy was included as a constant term only, while the eight beta coefficients and a random effect were included in the detection sub‐model. In all models, survey covariates consisting of continuous data were standardised (mean = 0, SD = 1) so that the relative importance of each covariate could be determined according to the magnitude of the coefficient (Schielzeth 2010). There were no strong correlations among the survey covariates within each model (Spearman's rank coefficient: |*r*_s_| < 0.6).

In the community‐level of the MSOM, hyper‐parameters (*μ*) were estimated that treated species‐level parameters as random effects drawn from a normal distribution (Zipkin, DeWan, and Andrew Royle 2009). For example, *β*_1*i*_ ~ *N*(*μ*_*β*1_, *σ*_*β*1_) where *μ*_*β*1_ is the mean community response (across species) to detection covariate 1, and *σ*_*β*1_ is the standard deviation in *β*_1_ among species. Thus the hyper‐parameters are simply the mean and variance for each covariate as measured across species (Zipkin, DeWan, and Andrew Royle 2009). Species' responses to survey covariates were assumed to be drawn from a common distribution where the species assessed have similar ecological requirements, and hence, species would have similar ecological responses to the covariates (Pacifici et al. 2014).

Model parameters and community summaries in MSOM were estimated using Bayesian inference with priors for the hyper‐parameters drawn from a normal distribution (*N*[0, 2.25]) and uniform priors for the standard deviation of the estimates (*U*[0, 5]) (Guillera‐Arroita, Kéry, and Lahoz‐Monfort 2019). In Model 5, priors for the constant terms were drawn from a beta distribution (beta[1, 1]), whereas priors for the other parameters were drawn from a uniform distribution (*U*[−5, 5]). A uniform prior was used to estimate the random effects in all five models (*U*[0, 5]). The means, standard deviations and the 2.5th and 97.5th percentiles of the posterior distributions of the model coefficients were estimated which represents 95% Bayesian credible intervals (BCI). Parameter estimates of covariates with a BCI that did not overlap zero were considered to represent the most influential relationships, whereas estimates with a BCI overlapping zero had greater uncertainty. However, some minor overlap of the BCI with zero was tolerated in inferring relationships; parameter estimates with ≥ 0.9 of the posterior distribution mass on one side of zero (either positive or negative) were also considered to be strongly influential (see Rowe et al. 2019). Covariates with a smaller standard deviation (*σ*) on hyper‐parameters were considered to have similar effects on detection across all amphibian species. Probabilities of detection and occupancy were derived using the inverse logit transformation of the intercept terms (*β*_0_ and *α*_0_, respectively).

Modelling was conducted using program JAGS (version 4.3.1, Plummer 2017) called via the R2jags package (Su and Yajima 2021) from R (version 4.3.0, R Core Team 2023). Each model was run using three replicate Markov chain Monte Carlo (MCMC) iterations to generate 350,000 samples from the posterior distribution of Models 1–4 (250,000 samples in Model 5) after discarding a ‘burn‐in’ of 50,000 samples (100,000 samples in Model 3) and a thinning rate of 3 (Models 2 and 3) or 5 (Models 1, 4 and 5). The Gelman‐Rubin statistic was checked for all estimated parameters which indicated acceptable convergence (i.e. $\hat{R}$ < 1.05; Brooks and Gelman 1998). Bayesian *p*‐values were calculated with the Freeman‐Tukey fit statistic to assess model fit (see Stolen et al. 2019). Values close to 0.5 indicated acceptable model fit (Gelman, Meng, and Stern 1996). We modelled predictive relationships for influential covariates while holding the other covariates in the model at their mean values.

## 3 Results

### 3.1 Detections

We detected nine native amphibian species during the surveys, with at least one species being detected at 83 ponds and no species detected at 17 ponds (Table 1). Larval and post‐metamorphic life stages were detected in all nine species while the eggs of five species were detected (Table A1). The *Pelophylax* spp. complex was detected most frequently (59 ponds; Table 1), while the European green toad (*Bufotes viridis*) and Danube crested newt (*Triturus dobrogicus*) were detected in only six and five ponds, respectively (Table 1). The common newt (*Lissotriton vulgaris*) and agile frog (*Rana dalmatina*) were each detected at 34 and 28 ponds, respectively (Table 1). The remaining four species were detected at between 12 and 21 ponds (Table 1). Fish were detected in 45 ponds including exotic species such as the eastern mosquitofish (*Gambusia holbrooki*) and common sunfish (*Lepomis gibbosus*; Table A2). The introduced goldfish (*Carassius auratus*) was the most frequently detected species (23 ponds; Table A2).

**TABLE 1:** Summary of model‐estimated probabilities of detection and occupancy for the amphibian community and nine species. Summaries are estimates using all three survey methods (call/visual encounter surveys, dip‐netting, newt trapping) simultaneously. Estimates were extracted from Model 1 and include 95% Bayesian credible intervals (BCI).

Species	No. of ponds detected	Estimated detection	95% BCI	Estimated occupancy	95% BCI
Community	83	0.320	0.142–0.598	0.461	0.271–0.674
Bombinatoridae					
*Bombina bombina*	12	0.032	0.001–0.331	0.393	0.173–0.765
Bufonidae					
*Bufo bufo*	16	0.152	0.011–0.699	0.350	0.179–0.644
*Bufotes viridis*	6	0.341	0.015–0.951	0.096	0.040–0.203
Hylidae					
*Hyla arborea*	17	0.118	0.010–0.551	0.452	0.236–0.769
Pelobatidae					
*Pelobates fuscus*	21	0.043	0.003–0.289	0.785	0.488–0.975
Ranidae					
*Pelophylax* spp. complex	59	0.571	0.220–0.891	0.798	0.635–0.933
*Rana dalmatina*	28	0.253	0.041–0.726	0.616	0.384–0.875
Salamandridae					
*Lissotriton vulgaris*	34	0.438	0.119–0.821	0.520	0.354–0.719
*Triturus dobrogicus*	5	0.088	0.002–0.695	0.129	0.043–0.348

Using Model 1, the mean probability of detecting an amphibian species during a single survey was 0.320 (95% BCI: 0.142–0.598; Table 1). Mean estimated probabilities of detection varied widely, and were highest for *Pelophylax* spp. (0.571, 0.220–0.891) and lowest for *Bombina bombina* (0.032, 0.001–0.331; Table 1). The mean probability of occupancy across the amphibian community was 0.461 (0.271–0.674), with individual estimates varying substantially (Table 1). The Bayesian *p*‐value was close to 0.5 indicating acceptable model fit (*p* = 0.449).

### 3.2 Community‐Level Detection

**FIGURE 2:**
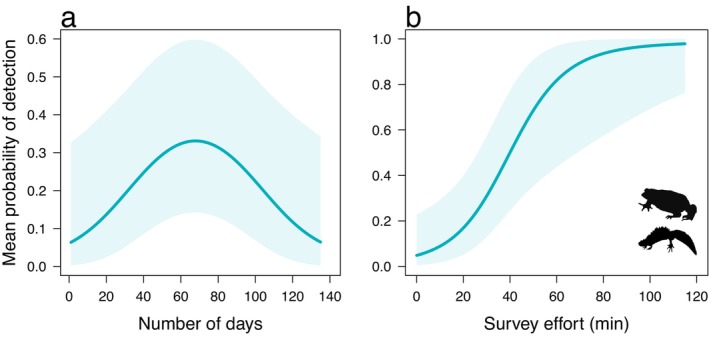
Mean estimates of the probability of detection (shaded areas are 95% Bayesian credible intervals) across the amphibian community versus: (a) number of days since 19 March 2023 (expressed as a quadratic relationship); and (b) survey effort represented by the number of minutes conducting call/visual encounter surveys.

There was a negative quadratic relationship between the mean estimated probability of detection and the number of days since 19 March (Table 2). Mean community detection was predicted to peak during survey 2 at 66–69 days (24–27 May; Figure 2). The relatively large standard deviation (*σ*) indicated that this relationship was variable among the nine species (Table 2). There was a positive relationship between the mean probability of detection and survey effort based on VES minutes, with a relatively small *σ* indicating similar responses by species to this covariate (Table 2). The predicted relationship indicated that at least 40 min of effort is required to attain ≥ 0.5 probability of detection (Figure 2). There were no influential relationships between the mean probabilities of detection and the remaining seven covariates (Table 2).

**TABLE 2:** Summary of hyper‐parameters for occupancy (*α*) and detection (*β*) covariates of the amphibian communities in central Hungary. Hyper‐parameter estimates were extracted from Model 1. Estimates include 95% Bayesian credible intervals (2.5th and 97.5th percentiles of the posterior distribution). Strongly influential relationships for beta estimates of the covariates are where ≥ 0.9 of the posterior weight of the mean estimate is above or below zero (highlighted in bold, except intercept coefficients).

Community‐level hyper‐parameter		Mean	SD	2.5th	97.5th
*μ*_*α*0_	Intercept	−0.158	0.434	−0.988	0.727
*σ*_*α*0_	Intercept	1.590	0.566	0.819	3.013
*μ*_*β*0_	Intercept	−0.752	0.556	−1.796	0.396
*σ*_*β*0_	Intercept	2.134	0.990	0.552	4.402
*μ*_*β*1_	Days	0.053	0.348	−0.649	0.739
*σ*_*β*1_	Days	0.451	0.400	0.016	1.487
***μ***_***β*2**_	**Days** ^**2**^	**−1.078**	**0.434**	**−1.886**	**−0.141**
*σ*_*β*2_	Days^2^	1.038	0.667	0.098	2.726
***μ***_***β*3**_	**Effort**	**1.789**	**0.528**	**0.480**	**2.660**
*σ*_*β*3_	Effort	0.845	0.900	0.023	3.509
*μ*_*β*4_	Temp	−0.136	0.413	−0.956	0.679
*σ*_*β*4_	Temp	1.084	0.516	0.344	2.346
*μ*_*β*5_	Water	0.162	0.280	−0.383	0.727
*σ*_*β*5_	Water	0.337	0.303	0.012	1.096
*μ*_*β*6_	Wind	−0.068	0.424	−0.903	0.782
*σ*_*β*6_	Wind	0.899	0.725	0.040	2.774
*μ*_*β*7_	Rain	−0.248	0.560	−1.335	0.865
*σ*_*β*7_	Rain	2.322	1.218	0.219	4.722
*μ*_*β*8_	Fish	−0.487	0.607	−1.667	0.722
*σ*_*β*8_	Fish	3.378	0.919	1.589	4.897
*μ*_*β*9_	Emergent	0.357	0.418	−0.534	1.141
*σ*_*β*9_	Emergent	1.242	0.699	0.281	3.031

*Note: μ* = beta estimate (mean community response); *σ* = standard deviation in the response to the covariate across species; SD = standard deviation. Days = number of days since 19 March 2023; Days^2^ = quadratic effect of Days; Effort = survey effort (number of minutes conducting call/visual encounter surveys); Temp = water temperature; Water = water levels (% of full water‐holding capacity at a pond); Wind = calm/light (0), moderate/strong (1); Rain = rainfall recorded during survey (1) or not (0); Fish = fish detected during a survey (1) or undetected (0); Emergent = % cover of emergent vegetation.

### 3.3 Species‐Specific Detection

**FIGURE 3:**
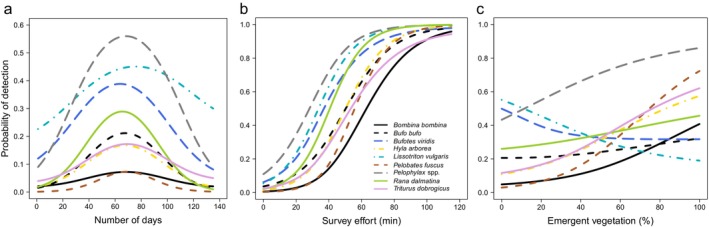
Species‐specific estimates of the probabilities of detection of nine amphibian species versus: (a) number of days (quadratic relationship); (b) survey effort; and (c) emergent vegetation cover. Credible intervals are omitted for clarity.

There were negative quadratic relationships between the estimated probabilities of detection of six species (*Bufo bufo*, *Bufotes viridis*, *Hyla arborea*, *Pelobates fuscus*, *Pelophylax* spp. and *R. dalmatina*) and the number of days, peaking at 63–67 days (Table 3; Figure 3).

**TABLE 3:** Summary of species‐specific estimates for occupancy (*α*) and detection (*β*) covariates for nine amphibian species. Estimates include 95% Bayesian credible intervals (2.5th and 97.5th percentiles of the posterior distribution). Parameter estimates were extracted from Model 1. Strongly influential relationships for beta estimates of the covariates are where ≥ 0.9 of the posterior weight of the mean estimate is above or below zero (highlighted in bold, except intercept coefficients).

Species	Species‐specific parameter		Mean	SD	2.5th	97.5th
*Bombina bombina*	*α*_0_	Intercept	−0.434	0.720	−1.566	1.182
	*β*_0_	Intercept	−3.398	1.595	−6.840	−0.703
	*β*_1_	Days	0.054	0.526	−1.046	1.113
	*β*_2_	Days^2^	−0.899	0.709	−2.277	0.578
	***β***_**3**_	**Effort**	**2.209**	**0.696**	**1.083**	**3.900**
	*β*_4_	Temp	−0.163	0.716	−1.615	1.254
	*β*_5_	Water	0.117	0.419	−0.777	0.943
	*β*_6_	Wind	−0.436	0.895	−2.564	1.107
	*β*_7_	Rain	−1.258	1.867	−5.519	2.043
	***β***_**8**_	**Fish**	**−2.392**	**1.845**	**−6.265**	**1.059**
	***β***_**9**_	**Emergent**	**0.976**	**0.670**	**−0.235**	**2.417**
*Bufo bufo*	*α*_0_	Intercept	−0.619	0.543	−1.520	0.591
	*β*_0_	Intercept	−1.718	1.333	−4.484	0.844
	*β*_1_	Days	0.063	0.533	−1.042	1.140
	***β***_**2**_	**Days** ^**2**^	**−1.719**	**0.740**	**−3.411**	**−0.519**
	***β***_**3**_	**Effort**	**1.862**	**0.547**	**0.771**	**2.952**
	*β*_4_	Temp	−0.363	0.722	−1.875	1.013
	*β*_5_	Water	0.175	0.412	−0.644	1.043
	*β*_6_	Wind	0.171	0.835	−1.315	2.107
	*β*_7_	Rain	0.613	1.481	−2.064	3.835
	*β*_8_	Fish	−1.557	1.542	−4.698	1.409
	*β*_9_	Emergent	0.139	0.688	−1.250	1.504
*Bufotes viridis*	*α*_0_	Intercept	−2.243	0.460	−3.176	−1.365
	*β*_0_	Intercept	−0.660	1.747	−4.172	2.958
	*β*_1_	Days	−0.198	0.687	−1.916	0.856
	***β***_**2**_	**Days** ^**2**^	**−1.408**	**0.908**	**−3.442**	**0.290**
	***β***_**3**_	**Effort**	**2.506**	**1.429**	**0.742**	**6.621**
	*β*_4_	Temp	0.023	0.969	−1.877	2.051
	*β*_5_	Water	0.261	0.527	−0.642	1.469
	*β*_6_	Wind	0.636	1.306	−1.107	4.068
	*β*_7_	Rain	−1.254	2.507	−7.133	3.261
	*β*_8_	Fish	1.733	2.165	−2.307	6.268
	*β*_9_	Emergent	−0.862	1.672	−4.929	1.620
*Hyla arborea*	*α*_0_	Intercept	−0.194	0.615	−1.173	1.204
	*β*_0_	Intercept	−2.010	1.226	−4.625	0.206
	*β*_1_	Days	0.036	0.524	−1.076	1.071
	***β***_**2**_	**Days** ^**2**^	**−1.384**	**0.680**	**−2.889**	**−0.148**
	***β***_**3**_	**Effort**	**2.187**	**0.612**	**1.161**	**3.613**
	*β*_4_	Temp	0.331	0.680	−0.943	1.764
	*β*_5_	Water	0.103	0.401	−0.754	0.877
	*β*_6_	Wind	0.013	0.796	−1.552	1.749
	***β***_**7**_	**Rain**	**−3.858**	**2.754**	**−10.164**	**0.092**
	***β***_**8**_	**Fish**	**−3.108**	**1.837**	**−6.856**	**0.367**
	***β***_**9**_	**Emergent**	**0.910**	**0.732**	**−0.370**	**2.545**
*Lissotriton vulgaris*	*α*_0_	Intercept	0.080	0.395	−0.600	0.941
	*β*_0_	Intercept	−0.250	0.887	−1.998	1.523
	*β*_1_	Days	0.210	0.499	−0.698	1.329
	*β*_2_	Days^2^	−0.488	0.607	−1.628	0.745
	***β***_**3**_	**Effort**	**2.106**	**0.568**	**1.121**	**3.385**
	*β*_4_	Temp	−0.717	0.632	−2.080	0.417
	*β*_5_	Water	0.109	0.377	−0.693	0.841
	*β*_6_	Wind	0.255	0.734	−1.012	1.935
	*β*_7_	Rain	−0.955	1.282	−3.725	1.411
	***β***_**8**_	**Fish**	**−3.013**	**1.621**	**−6.203**	**0.248**
	*β*_9_	Emergent	−0.654	0.620	−1.956	0.497
*Pelobates fuscus*	*α*_0_	Intercept	1.298	0.951	−0.047	3.654
	*β*_0_	Intercept	−3.091	1.224	−5.671	−0.899
	*β*_1_	Days	0.225	0.560	−0.755	1.519
	***β***_**2**_	**Days** ^**2**^	**−2.406**	**0.969**	**−4.629**	**−0.928**
	***β***_**3**_	**Effort**	**2.626**	**0.804**	**1.451**	**4.562**
	*β*_4_	Temp	−0.200	0.694	−1.653	1.121
	*β*_5_	Water	0.115	0.388	−0.706	0.879
	*β*_6_	Wind	0.190	0.796	−1.233	2.009
	*β*_7_	Rain	1.143	1.543	−1.463	4.520
	***β***_**8**_	**Fish**	**−5.799**	**2.214**	**−10.678**	**−2.069**
	***β***_**9**_	**Emergent**	**1.589**	**0.649**	**0.475**	**2.999**
*Pelophylax* spp. complex	*α*_0_	Intercept	1.372	0.552	0.553	2.639
	*β*_0_	Intercept	0.287	0.849	−1.267	2.099
	*β*_1_	Days	0.085	0.427	−0.781	0.949
	***β***_**2**_	**Days** ^**2**^	**−1.284**	**0.498**	**−2.360**	**−0.400**
	***β***_**3**_	**Effort**	**1.892**	**0.489**	**0.960**	**2.887**
	***β***_**4**_	**Temp**	**1.096**	**0.589**	**0.029**	**2.354**
	*β*_5_	Water	0.326	0.372	−0.313	1.174
	*β*_6_	Wind	−0.492	0.660	−1.978	0.656
	*β*_7_	Rain	−1.056	1.055	−3.334	0.842
	***β***_**8**_	**Fish**	**2.507**	**1.087**	**0.563**	**4.817**
	***β***_**9**_	**Emergent**	**0.751**	**0.439**	**−0.036**	**1.696**
*Rana dalmatina*	*α*_0_	Intercept	0.474	0.643	−0.474	1.950
	*β*_0_	Intercept	−1.085	1.031	−3.152	0.976
	*β*_1_	Days	−0.114	0.569	−1.432	0.878
	***β***_**2**_	**Days** ^**2**^	**−1.918**	**0.763**	**−3.690**	**−0.737**
	***β***_**3**_	**Effort**	**2.421**	**0.661**	**1.382**	**3.968**
	***β***_**4**_	**Temp**	**−1.383**	**0.788**	**−3.095**	**0.004**
	*β*_5_	Water	0.191	0.388	−0.565	1.014
	*β*_6_	Wind	−0.492	0.773	−2.308	0.810
	*β*_7_	Rain	0.748	1.454	−1.804	3.936
	***β***_**8**_	**Fish**	**−3.172**	**1.538**	**−6.479**	**−0.428**
	*β*_9_	Emergent	0.290	0.513	−0.746	1.308
*Triturus dobrogicus*	*α*_0_	Intercept	−1.909	0.630	−3.103	−0.627
	*β*_0_	Intercept	−2.334	1.821	−6.414	0.824
	*β*_1_	Days	0.132	0.599	−1.024	1.446
	*β*_2_	Days^2^	−1.179	0.876	−3.017	0.597
	***β***_**3**_	**Effort**	**2.141**	**0.930**	**0.502**	**4.434**
	*β*_4_	Temp	−0.289	0.880	−2.122	1.418
	*β*_5_	Water	0.130	0.476	−0.891	1.072
	*β*_6_	Wind	−0.537	1.111	−3.334	1.228
	*β*_7_	Rain	0.479	1.973	−3.143	4.900
	*β*_8_	Fish	−1.319	2.821	−6.630	4.829
	***β***_**9**_	**Emergent**	**1.164**	**0.967**	**−0.510**	**3.337**

*Note:* See Table 2 for a description of the parameters.

Abbreviation: SD = standard deviation.

There was a positive relationship between the estimated probability of detection of all nine species and survey effort based on VES minutes (Table 3; Figure 3). The survey effort required to achieve a ≥ 0.5 probability of detection ranged from 27 min (*Pelophylax* spp.) to 62 min (*Bombina bombina*; Figure 3), with an average of 45 min across the nine species.

There was a positive relationship between the probability of detecting *Pelophylax* spp. and water temperature, whereas there was a negative relationship for *R. dalmatina* (Table 3). There was a negative relationship between the probability of detection of *H. arborea* and rain (Table 3). There were no influential relationships with water levels or wind (Table 3).

**FIGURE 4:**
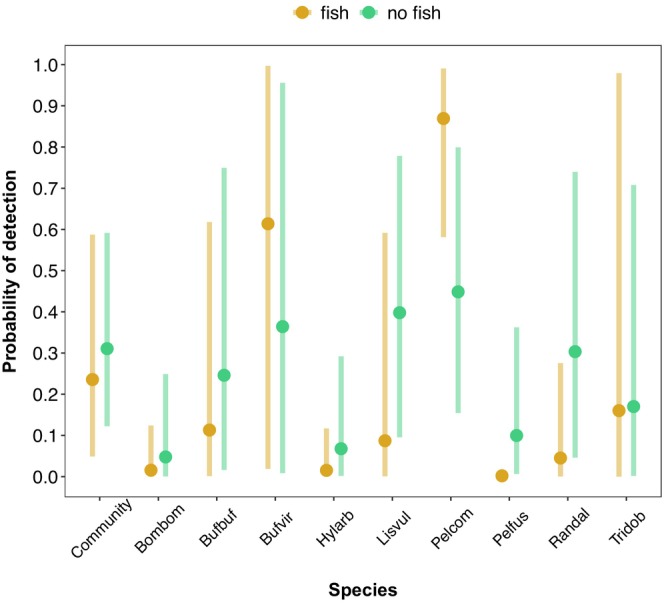
Predicted estimates (vertical bars are 95% Bayesian credible intervals) of the probabilities of detection of the amphibian community and nine species versus detection/non‐detection of fish at ponds. Species codes: Bombom = *Bombina bombina*; Bufbuf = *Bufo bufo*; Bufvir = *Bufotes viridis*; Hylarb = *Hyla arborea*; Lisvul = *Lissotriton vulgaris*; Pelfus = *Pelobates fuscus*; Pelcom = *Pelophylax* spp. complex; Randal = *Rana dalmatina*; Tridob = *Triturus dobrogicus*.

There were positive relationships between emergent vegetation cover and the probability of detecting *Bombina bombina*, *H. arborea*, *Pelobates fuscus*, *Pelophylax* spp. and *T. dobrogicus* (Table 3; Figure 3). There were negative relationships between the probability of detecting *Bombina bombina*, *H. arborea*, *L. vulgaris*, *Pelobates fuscus* and *R. dalmatina*, and the detection of fish, although there was a positive relationship between *Pelophylax* spp. and fish (Table 3; Figure 4).

### 3.4 Survey Methods

**FIGURE 5:**
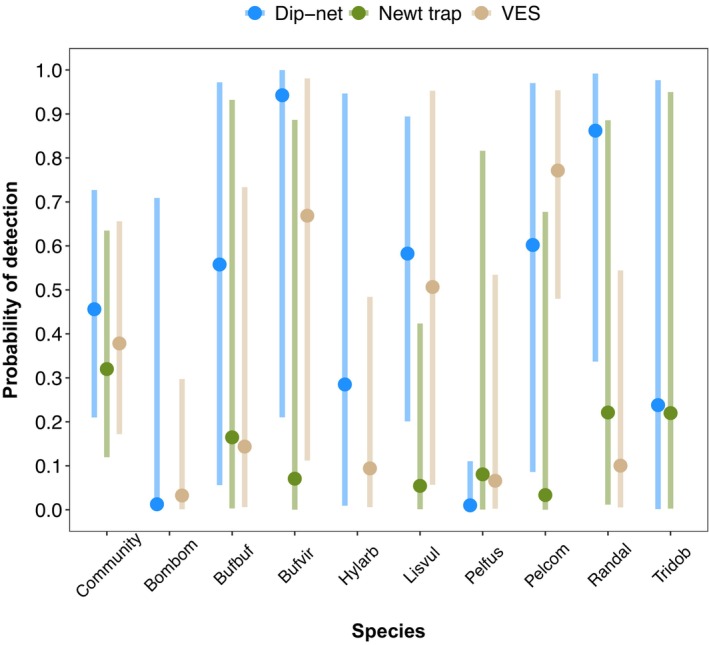
Predicted estimates (vertical bars are 95% Bayesian credible intervals) of the probabilities of detection of the amphibian community and nine species using three survey methods: Call/visual encounter surveys (VES), dip‐netting and newt traps. See Figure 4 for an explanation of the species codes.

Models 2–4 included detection data from 100, 98 and 41 sites obtained using each respective survey method, and included Days, Days^2^ and Effort as survey covariates. There was no substantial difference in the mean probability of detection among the three methods, with very wide and overlapping 95% BCIs, although mean community detection was highest using dip‐netting (Figure 5). The probabilities of detecting *Bufo bufo*, *Bufotes viridis*, *L. vulgaris*, *Pelobates fuscus* and *R. dalmatina* with dip‐nets were 2–12 times higher than using traps (Figure 5). The probabilities of detection using VES, dip‐netting and traps were highest for *Pelophylax* spp., *Bufotes viridis* and *R. dalmatina*, respectively (Figure 5). All nine species were detected by dip‐netting whereas *T. dobrogicus* was not detected using VES, and *Bombina bombina* and *H. arborea* were not detected in newt traps (Figure 5). The only post‐metamorphic anurans trapped were an adult *Pelobates fuscus* and juvenile *Pelophylax* spp.

**FIGURE A1:**
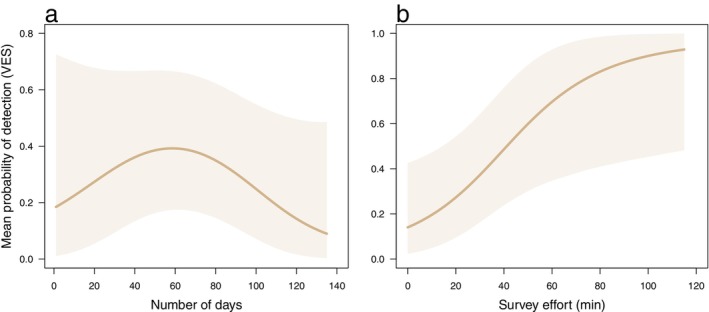
Mean estimates of the probability of detection during call/ visual encounter surveys (shaded areas are 95% Bayesian credible intervals) across the amphibian community versus: (a) number of days since 19 March 2023 (expressed as a quadratic relationship); (b) survey effort (number of minutes spent surveying).

**FIGURE A2:**
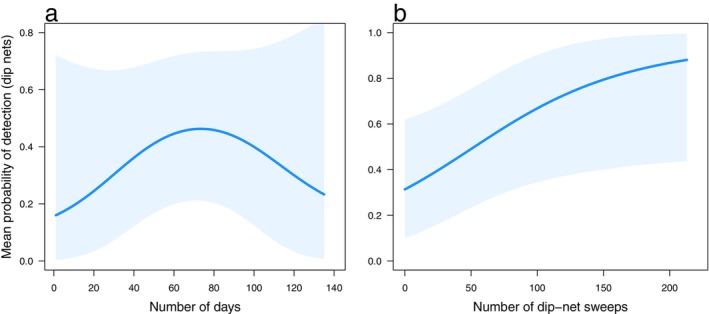
Mean estimates of the probability of detection during dip‐netting surveys (shaded areas are 95% Bayesian credible intervals) across the amphibian community versus: (a) number of days since 19 March 2023 (expressed as a quadratic relationship); (b) survey effort (number of dip‐net sweeps).

At the community level, there were influential relationships between the mean probability of detection using VES and dip‐netting, and Days^2^ and Effort (Table S2, Figures A1 and A2). There were no influential relationships between the mean probability of detection using newt traps and either number of days or number of traps (Table S2).

**FIGURE A3:**
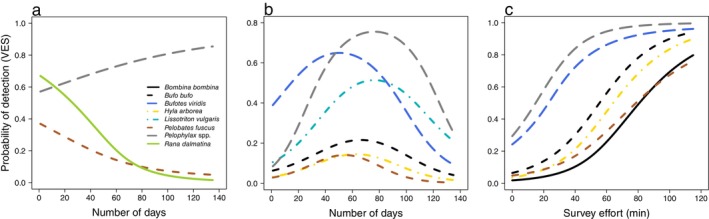
Species‐specific estimates of the probabilities of detection of eight amphibian species during call/ visual encounter surveys versus: (a) number of days since 19 March 2023; (b) number of days expressed as a quadratic relationship; and (c) survey effort (number of minutes spent surveying). Credible intervals here and in Figs. A4 and A5 are omitted for clarity.

**FIGURE A4:**
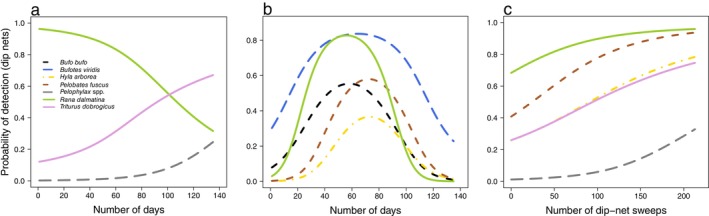
Species‐specific estimates of the probabilities of detection of seven amphibian species during dip‐netting surveys versus: (a) number of days since 19 March 2023; (b) number of days expressed as a quadratic relationship; and (c) survey effort (number of dip‐net sweeps).

**FIGURE A5:**
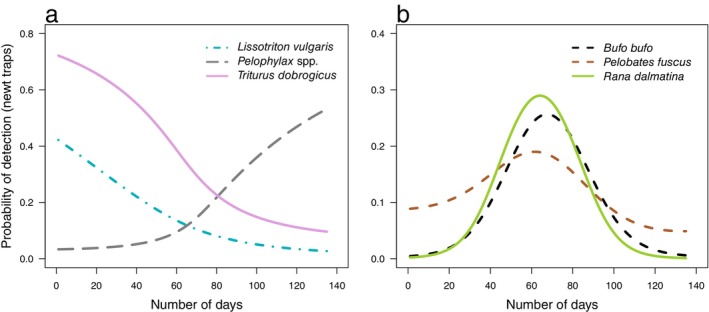
Species‐specific estimates of the probabilities of detection of six amphibian species during newt‐trapping surveys versus: (a) number of days since 19 March 2023; and (b) number of days expressed as a quadratic relationship.

There was a linear decrease in detection probabilities using VES with number of days for *Pelobates fuscus* and *R. dalmatina*, while detection increased for *Pelophylax* spp. (Table S3; Figure A3). There were negative quadratic relationships using VES, dip‐netting and newt traps, and number of days for six, five and three species, respectively (Table S3; Figures A3, A4, A5). There were linear decreases in detection probabilities with number of days using dip‐netting (*R. dalmatina*) and traps (*L. vulgaris* and *T. dobrogicus*), whereas detection of *Pelophylax* spp. increased (Table S3, Figures A4 and A5). The probability of detecting *T. dobrogicus* using dip‐netting increased with number of days (Table S3, Figure A4).

The probability of detection increased with the number of minutes spent conducting VES for six species (Figure A3), while detection increased with the number of dip‐net sweeps for five species (Table S3, Figure A4).

### 3.5 Detection of Fish

**FIGURE 6:**
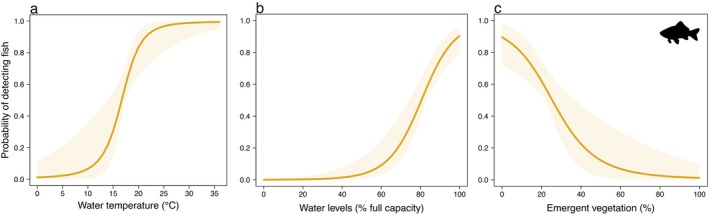
Predicted estimates of the probability of detecting fish at a pond (shaded areas are 95% Bayesian credible intervals) versus: (a) water temperature; (b) water levels; and (c) emergent vegetation cover.

The mean probability of detecting fish during a single survey was 0.588 (95% BCI: 0.503–0.717) while the mean estimated probability of occupancy was 0.601 (0.509–0.707). There were positive relationships between the probability of detection and both water temperature and water levels (Table 4; Figure 6). There was a negative relationship between the probability of detecting fish and emergent vegetation cover (Table 4; Figure 6). There was no influential relationship between the probability of detection and number of days (Table 4). The Bayesian *p*‐value indicated acceptable model fit (*p* = 0.379).

**TABLE 4:** Estimates of occupancy (*α*) and detection (*β*) covariates for fish. Estimates include 95% Bayesian credible intervals (2.5th and 97.5th percentiles of the posterior distribution). Strongly influential relationships for beta estimates of the covariates are where ≥ 0.9 of the posterior weight of the mean estimate is above or below zero (highlighted in bold, except intercept coefficients). Estimates include all fish species detected during the surveys.

Parameter		Mean	SD	2.5th	97.5th
*α*_0_	Intercept	0.408	0.225	0.036	0.879
*β*_0_	Intercept	0.355	0.264	0.012	0.930
*β*_1_	Days	−0.039	1.128	−2.075	2.280
*β*_2_	Days^2^	0.627	0.576	−0.460	1.809
*β*_3_	Effort	0.507	0.620	−0.686	1.753
***β***_**4**_	**Temp**	**3.041**	**1.089**	**0.824**	**4.852**
***β***_**5**_	**Water**	**3.908**	**0.757**	**2.184**	**4.951**
*β*_6_	Wind	−1.353	1.073	−3.445	0.777
*β*_7_	Rain	−1.281	1.545	−4.202	1.881
***β***_**8**_	**Emergent**	**−2.487**	**0.837**	**−4.154**	**−0.878**

*Note:* See Table 2 for a description of the parameters.

Abbreviation: SD = standard deviation.

## 4 Discussion

Accounting for the imperfect detection of species in occupancy models is crucial for understanding relationships between amphibian communities and the presence of predatory fish (Hamer, Schmera, and Mahony 2021; Rowe et al. 2019). We conducted repeated surveys at ponds using three methods (call/visual encounter surveys, dip‐netting and newt trapping) to assess amphibian and fish detection probabilities. We found highly variable detection rates of amphibians and relatively low detection probabilities of some species, with distinct peaks in species detectability over time that differed according to survey method. The probability of detecting fish at a pond was much higher and fish decreased the probability of detecting amphibians. A high cover of emergent vegetation at a pond increased and decreased the probability of detecting amphibians and fish, respectively. Overall, we found that a high survey effort was required to detect amphibian species and that dip‐net surveys provided a slightly higher chance of detecting amphibians if conducted during optimal periods.

Mean community detection was predicted to peak in late May, when both larvae and adults of multiple species were active and available for detection. However, there was considerable interspecific variation in peak detection rates due to differences in reproductive strategies and breeding season (Wells 1977). These differences became more apparent among the three survey methods used. For example, explosive‐breeding species such as *Bufo bufo* and *Rana dalmatina* showed early spring peaks in detection using dip‐netting compared with prolonged, summer‐breeding *Pelophylax* spp. detected during VES. Detection rates of *R. dalmatina* using VES were also higher in early spring, when egg masses were observed in ponds. Although we did not analyse each life stage separately, these patterns were likely correlated with specific life stages being better detected using a particular survey method. For example, we only captured adult *Triturus dobrogicus* in newt traps in early spring, whereas larvae were caught in dip‐nets in late spring and summer. It therefore appears that while newt trapping had a relatively low probability of mean detection across the community – with non‐detections of two species – it was the most suitable method for detecting adult *T. dobrogicus* in ponds. Dewsbury newt traps were designed specifically for the safe capture of adult *Triturus* spp. (Dewsbury 2014), although dip‐netting is still an effective survey method for this species (Arntzen 2002). We also trapped post‐metamorphic *Pelobates fuscus* and *Pelophylax* spp., perhaps because male *Pelobates fuscus* call underwater while *Pelophylax* spp. are largely aquatic.

A combination of survey methods is generally considered to yield higher detection probabilities for amphibians than using single techniques (Nichols et al. 2008; Petitot et al. 2014; Sewell, Beebee, and Griffiths 2010). Importantly, the use of multiple methods together with a relatively high survey effort was required for us to achieve comparatively modest detection probabilities during a single survey. Greater survey effort, including time spent searching or trap effort, typically results in higher detection rates for many amphibian species (e.g. Canessa et al. 2012; Sewell, Beebee, and Griffiths 2010). We found relatively low detection rates during a single survey for three of the nine species detected (e.g. *Bombina bombina*). Nonetheless, *Bombina bombina* was not necessarily rare in the study area, with the probability of occupancy estimated to be three times higher than the naïve occupancy rate. Conversely, occupancy estimates for *Bufotes viridis* and *T. dobrogicus* were very low, even with a relatively high probability of detection for *Bufotes viridis*. We also found no discernible relationship between detection rates and the number of newt traps deployed, but we used only a small range of traps (2–4 per pond) that may limit our ability to make confident inferences on trap effort and detection probabilities. Our results confirm that studies into species‐habitat relationships that do not account for low detection rates with high variability among species through the sampling season will likely be subject to bias if variations in detection probabilities are not considered (Baumgardt et al. 2021; Petitot et al. 2014).

We combined all life stages in modelling detection probabilities due to the multiple methods we used. However, under certain circumstances, there may be large differences between larval and adult detection probabilities of amphibians because different methods are often used to survey each life stage (Wassens, Hall, and Spencer 2017). Furthermore, the detection of any life stage at a pond provides inference on habitat use, but is not indicative of successful reproduction as the presence of adult amphibians may not result in breeding (Crawford et al. 2023; Wassens, Hall, and Spencer 2017). Multi‐state occupancy models estimate both occupancy and breeding probabilities contingent on overall species' detection probabilities and breeding life stages (i.e. eggs or larvae), and may provide greater insight into amphibian occupancy dynamics (Cruickshank, Bergamini, and Schmidt 2021). Otherwise, we suggest differentiating between life stages when modelling metacommunity dynamics in the study area, using detection of larvae and post‐metamorphic life stages as separate response variables in occupancy models.

There were negative relationships between the probabilities of detection of five species (*Bombina bombina*, *H. arborea*, *L. vulgaris*, *Pelobates fuscus* and *R. dalmatina*) and the detection of fish at a pond. Although fish species were grouped together, many of the species we detected can have detrimental effects on amphibian species due to predation or non‐lethal injuries, or altered activity levels (e.g. goldfish; Monello and Wright 2001). Goldfish presence in ponds can cause newts (e.g. *Lissotriton* spp.) to leave the water (Winandy, Darnet, and Denoël 2015), and therefore be unavailable for detection during aquatic surveys, or reduce activity levels and increase shelter usage in other newt species (Winandy and Denoël 2013). Topmouth gudgeon (*Pseudorasbora parva*) were also frequently detected, which can reduce survival rates of larval *H. arborea* and *R. dalmatina*, as well as increase refuge use and reduce activity rates in *R. dalmatina* (Teplitsky, Plénet, and Joly 2003). Other frequently detected non‐native fish species (e.g. common sunfish) reduce occupancy rates of *H. arborea* at ponds and consume *Triturus* spp. larvae (Préau et al. 2017). In contrast, there was a positive relationship between the detection of *Pelophylax* spp. and fish. The larvae of *Pelophylax* spp. are less vulnerable to fish predation than *H. arborea* and *R. dalmatina*, and often co‐occur in ponds with fish because they modify their behaviour in response to predatory fish (Hartel et al. 2007; Teplitsky, Plénet, and Joly 2003). Separating fish into predatory and non‐predatory species in future studies may provide a clearer picture of the effects of fish on amphibian occupancy.

Infrequently detected species such as *Bombina bombina* and *Pelobates fuscus* are usually associated with fishless ponds (Kloskowski, Nieoczym, and Stryjecki 2020), and so high occupancy rates of fish at ponds are likely reducing their detectability in the study area. For example, in a previous study, there was a negative relationship between the abundance of *Bombina bombina* and *Pelobates fuscus* larvae and the presence of fish (see Hamer, Barta, et al. 2021). However, in this study, high emergent vegetation cover at ponds increased the probability of detecting five species, including rarely‐detected species. Emergent vegetation in ponds increases aquatic habitat complexity and provides refugia that amphibians can use to evade predatory fish (Hartel et al. 2007). Besides the effects of fish, occupancy rates of infrequently detected species may be relatively low in the study area due to roads and urbanisation reducing abundance (Hamer, Barta, et al. 2021; Tóth‐Ronkay et al. 2015; Vörös, Kiss, and Puky 2015).

The probability of detecting fish was lower in ponds with high emergent vegetation cover (where amphibian detection was higher) and low water levels, potentially making it difficult to uncouple the effects of fish, vegetation and hydroperiod on amphibian occupancy. For instance, studies might not find a positive interaction between emergent vegetation and amphibian occupancy when fish are present, but the apparent lack of an association might be false due to low fish detection rates at ponds with high vegetation cover. It is therefore crucial that studies at ponds investigating relationships between amphibian occupancy and fish account for imperfect detection of both amphibian and fish species. For example, some studies of amphibian occupancy acknowledged poor detection rates of fish that may be due to sampling bias, because traps placed at the shallow margins of ponds were unable to capture fish occurring in deeper‐water microhabitats where traps were not set (Rowe et al. 2019). We also restricted our dip‐netting and trapping to relatively shallow water depths (< 1.2 m). However, low fish detection rates at ponds with low water levels may reflect true absences, given that 16 ponds dried out completely during the study, potentially eliminating any fish present. Heterogeneity in fish detection probabilities may therefore reduce the ability to determine impacts of non‐native predators on amphibian occupancy, especially local extinctions (Adams et al. 2011). Accordingly, we recommend including detection‐error corrected estimates of fish presence at each site in amphibian occupancy models that examine relationships between amphibian distribution and predatory fish.

**FIGURE 7:**
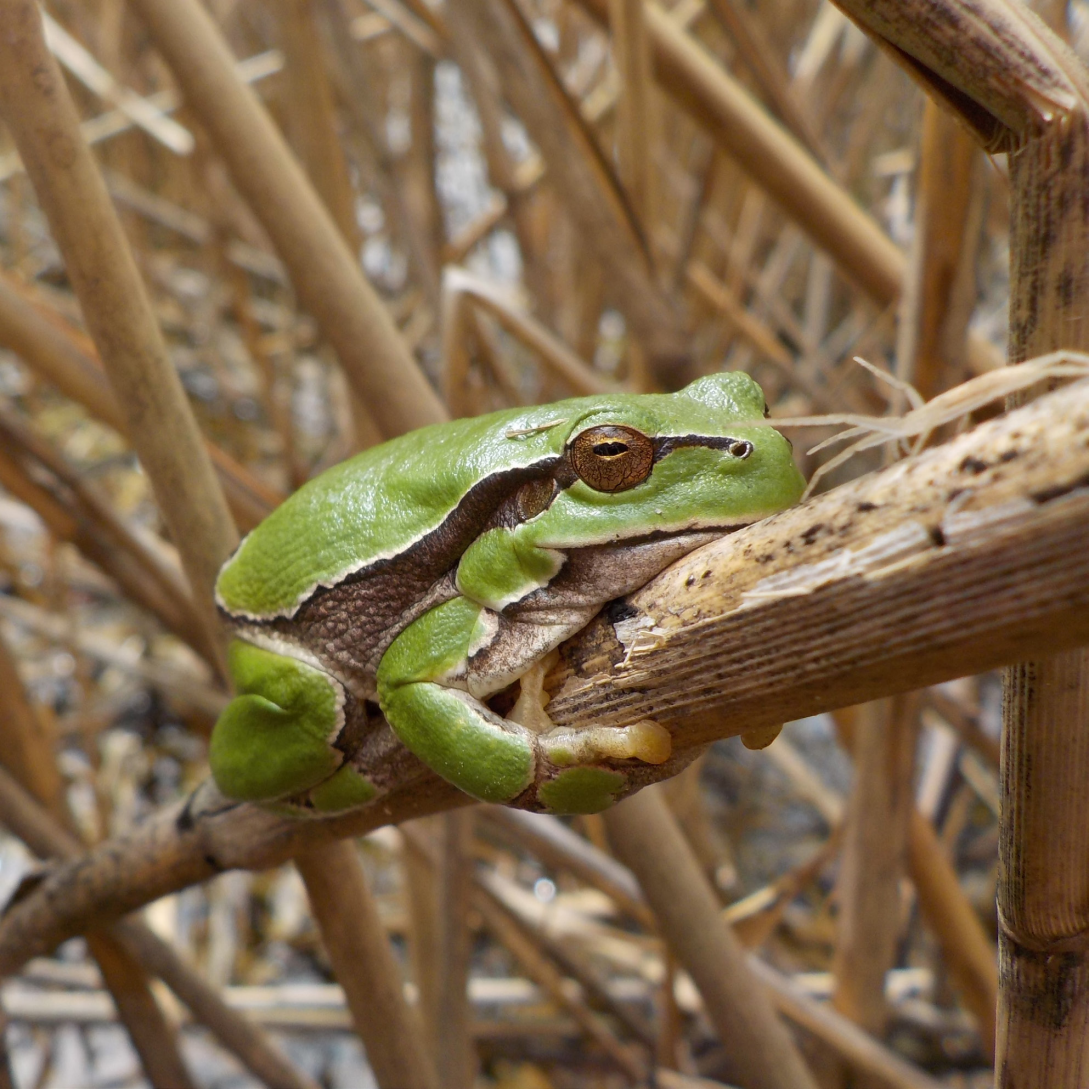
European tree frog (*Hyla arborea*) basking in sunlight. We rarely detected this species at ponds with fish.

There were influential relationships between detection probabilities of several amphibian species and climatic variables. For example, detection rates of *Pelophylax* spp. increased with warmer water temperatures, likely because of increased detection probability during summer when this species is more active (Berninghausen and Berninghausen 2001). Conversely, detection of *R. dalmatina* was higher at cooler water temperatures, which occur more often in early spring when this species spawns (Arnold and Ovenden 2002). The probability of detecting fish increased with higher water temperatures, possibly because of increased swimming activity (e.g. tail‐beat frequency; Bartolini, Butail, and Porfiri 2015), and hence visibility and trappability. Surprisingly, there was a negative relationship between the probability of detecting *H. arborea* and rain, when calling activity usually increases (Berninghausen and Berninghausen 2001). This relationship is likely because we detected all adult *H. arborea* basking in sunlight (see Figure 7), while rain would otherwise indicate overcast conditions.

In conclusion, we found that detection probabilities of amphibian species in the study area were highly variable although the probability of detecting fish was generally much higher. As our sampling effort of all amphibian life stages was relatively intense and we detected all nine native pond‐breeding species known to occur in Budapest (Tóth‐Ronkay et al. 2015), we do not recommend increasing the number of surveys in an effort to improve the detectability of rare species at sites. Rather, we consider it crucial that freshwater community studies apply multi‐species occupancy models that account for both the imperfect detection of species, and share data across the community to increase the precision of occupancy estimates for rare species (Guzy et al. 2019; Zipkin, DeWan, and Andrew Royle 2009). This approach will be ideal for assessing the impact of introduced fish and other environmental stressors (e.g. urbanisation) at both community and species levels.

## Author Contributions

**Andrew J. Hamer:** conceptualization (lead), data curation (lead), formal analysis (lead), funding acquisition (lead), investigation (equal), methodology (lead), project administration (equal), resources (lead), supervision (lead), validation (lead), visualization (lead), writing – original draft (lead), writing – review and editing (lead). **Júlia Horányi:** investigation (equal), project administration (equal), resources (supporting), writing – review and editing (supporting).

## Conflicts of Interest

The authors declare no conflicts of interest.

## Supporting information


Table S1
Table S2
Table S3

## Data Availability

Data and model code that support the findings of this study are available from the corresponding author upon reasonable request. Additional data are available in the Supporting Information and can be accessed at this doi: https://doi.org/10.5061/dryad.70rxwdc6w.

## Appendix 1

**TABLE A1:** Detection (●) and non‐detection (○) of each life stage of nine amphibian species using three survey methods (VES: call/visual encounter surveys; dip‐netting and newt traps) at ponds in central Hungary. Images correspond to eggs (●), larvae and post‐metamorphic life stages, respectively.

Species				
*Bombina bombina*	●	○	○	○
		○	●	○
		●	●	○
*Bufo bufo*	●	●	●	○
		●	●	●
		●	●	○
*Bufotes viridis*	●	●	●	○
		●	●	●
		●	○	○
*Hyla arborea*	●	○	○	○
		●	●	○
		●	○	○
*Lissotriton vulgaris*	●	○	○	○
		●	●	●
		●	●	●
*Pelobates fuscus*	●	●	●	○
		●	●	●
		●	○	●
*Pelophylax* spp. complex	●	●	●	○
		●	●	●
		●	●	●
*Rana dalmatina*	●	●	●	○
		●	●	●
		●	●	○
*Triturus dobrogicus*	●	○	○	○
		○	●	○
		○	○	●

*Note*: Silhouette images of amphibians taken from PhyloPic v2.0 (www.phylopic.org) and are public domain images. Anuran larvae were defined as individuals within Gosner development stages 25 (small tadpoles large enough to be reliably identified) through stages 42–44 (metamorphosing tadpoles with front and hind limbs; Gosner, 1960). Newt larvae were identified using similar morphological parameters (stages 39–55; Gallien and Bidaud, 1959).

**References** Gallien, L., Bidaud, O., 1959. Table chronologique du développement chez *Triturus helveticus* Razoumowsky. *Bulletin de la Société zoologique de France* 84, 22–32. Gosner, K.L., 1960. A simplified table for staging anuran embryos and larvae with notes on identification. *Herpetologica* 16, 183–190.

**TABLE A2:** Detection (●) and non‐detection (○) of fish species using three survey methods (VES: visual encounter surveys; dip‐netting and newt traps) at ponds in central Hungary.

Species	Common name	No. of sites	Proportion of sites	Detection method		
		
*Carassius auratus* ^a^	Goldfish	23	0.23	●	●	○
*Lepomis gibbosus* ^a^	Common sunfish	13	0.13	●	●	●
*Cyprinus rubrofuscus* ^a^	Amur/Koi carp	12	0.12	●	●	○
Unknown spp.^b^	Unknown spp.	11	0.11	●	○	○
*Cyprinus carpio*	Common carp	8	0.08	●	○	○
*Pseudorasbora parva* ^a^	Topmouth gudgeon	7	0.07	●	●	●
*Ameiurus melas* ^a^	Black bullhead	5	0.05	●	●	●
*Leucaspius delineatus*	Sunbleak	5	0.05	○	●	●
*Perca fluviatilis*	European perch	5	0.05	●	●	●
*Gambusia holbrooki* ^a^	Eastern mosquitofish	4	0.04	●	●	○
*Carassius gibelio*	Prussian carp	3	0.03	●	●	○
*Esox lucius*	Northern pike	3	0.03	○	●	○
*Scardinius erythrophthalmus*	Common rudd	3	0.03	●	○	●
*Poecilia reticulata* ^a^	Guppy	2	0.02	●	●	○
*Chondrostoma nasus*	Common nase	1	0.01	●	○	○
*Hemichromis guttatus* ^a^	Spotted jewelfish	1	0.01	○	●	○
*Misgurnus fossilis*	Weatherfish	1	0.01	○	○	●
*Poecilia sphenops* ^a^	Black molly	1	0.01	●	○	○
*Proterorhinus semilunaris* ^a^	Western tubenose goby	1	0.01	○	●	○
*Rutilus rutilus*	Common roach	1	0.01	●	○	○
*Xiphophorus maculatus* ^a^	Red wagtail platy	1	0.01	●	○	○

^a^ Invasive (non‐native) species in Hungary (see Haraszthy L. (Ed.), (2022): Invasive Animal Species in Hungary. Duna–Ipoly National Park Directorate—Ministry of Foreign Affairs and Trade of Hungary, Budapest. /Rosalia Handbooks 5./)

^b^ Unknown species seen during surveys (VES) and unable to be reliably identified.

## References

[ece370383-bib-0001] Adams, M. J. , C. A. Pearl , S. Galvan , and B. McCreary . 2011. “Non‐Native Species Impacts on Pond Occupancy by an Anuran.” Journal of Wildlife Management 75: 30–35. 10.1002/jwmg.29.

[ece370383-bib-0002] Adams, M. J. , K. O. Richter , and W. P. Leonard . 1997. “Surveying and Monitoring Amphibians Using Aquatic Funnel Traps.” In Sampling Amphibians in Lentic Habitats, Northwest Fauna Number 4, edited by D. H. Olson , W. P. Leonard , and R. B. Bury , 47–54. Washington, DC: Society for Northwestern Vertebrate Biology, Olympia.

[ece370383-bib-0003] Alford, R. A. , and S. J. Richards . 1999. “Global Amphibian Declines: A Problem in Applied Ecology.” Annual Review of Ecology and Systematics 30: 133–165.

[ece370383-bib-0004] Amburgey, S. M. , L. L. Bailey , M. Murphy , E. Muths , and W. C. Funk . 2014. “The Effects of Hydropattern and Predator Communities on Amphibian Occupancy.” Canadian Journal of Zoology 92: 927–937. 10.1139/cjz-2014-0106.

[ece370383-bib-0005] Arnold, N. , and D. Ovenden . 2002. A Field Guide to the Reptiles and Amphibians of Britain and Europe. 2nd ed. London, UK: HarperCollinsPublishers Ltd. .

[ece370383-bib-0006] Arntzen, J. W. 2002. “Testing for Equal Catchability of *Triturus* Newts by Dip Netting.” Journal of Herpetology 36: 272–276. 10.1670/0022-1511(2002)036[0272:TFECOT]2.0.CO;2.

[ece370383-bib-0007] Bailey, L. L. , T. R. Simons , and K. H. Pollock . 2004. “Estimating Site Occupancy and Species Detection Probability Parameters for Terrestrial Salamanders.” Ecological Applications 14: 692–702.

[ece370383-bib-0008] Bartolini, T. , S. Butail , and M. Porfiri . 2015. “Temperature Influences Sociality and Activity of Freshwater Fish.” Environmental Biology of Fishes 98: 825–832. 10.1007/s10641-014-0318-8.

[ece370383-bib-0009] Baumgardt, J. A. , M. L. Morrison , L. A. Brennan , M. Thornley , and T. A. Campbell . 2021. “Variation in Herpetofauna Detection Probabilities: Implications for Study Design.” Environmental Monitoring and Assessment 193: 658. 10.1007/s10661-021-09424-0. 34533627 PMC8448696

[ece370383-bib-0010] Berninghausen, O. , and F. Berninghausen . 2001. Whose Tadpole Is It? The Waterproof Field Guide to Central European Amphibians. Hannover, Germany: NABU Germany (German Association for the Protection of Nature).

[ece370383-bib-0011] Brooks, S. P. , and A. Gelman . 1998. “General Methods for Monitoring Convergence of Iterative Simulations.” Journal of Computational and Graphical Statistics 7: 434–455. 10.1080/10618600.1998.10474787.

[ece370383-bib-0012] Canessa, S. , G. W. Heard , K. M. Parris , and M. A. McCarthy . 2012. “Integrating Variability in Detection Probabilities When Designing Wildlife Surveys: A Case Study of Amphibians From South‐Eastern Australia.” Biodiversity and Conservation 21: 729–744. 10.1007/s10531-011-0211-0.

[ece370383-bib-0013] Crawford, J. A. , W. E. Peterman , A. R. Kuhns , and C. A. Phillips . 2023. “Effectiveness of Rapid Sampling Assessments for Wetland‐Breeding Amphibians.” Ecological Indicators 154: 110736. 10.1016/j.ecolind.2023.110736.

[ece370383-bib-0014] Cruickshank, S. S. , A. Bergamini , and B. R. Schmidt . 2021. “Estimation of Breeding Probability Can Make Monitoring Data More Revealing: A Case Study of Amphibians.” Ecological Applications 31: e02357. 10.1002/eap.2357. 33870588

[ece370383-bib-0015] Crump, M. L. , and N. J. Scott Jr . 1994. “Visual Encounter Surveys.” In Measuring and Monitoring Biological Diversity. Standard Methods for Amphibians, edited by W. R. Heyer , M. A. Donnelly , R. W. McDiarmid , L. C. Hayek , and M. S. Foster , 84–92. Washington, DC: Smithsonian Institution Press.

[ece370383-bib-0016] Curtis, A. E. , and P. W. C. Paton . 2010. “Assessing Detection Probabilities of Larval Amphibians and Macroinvertebrates in Isolated Ponds.” Wetlands 30: 901–914. 10.1007/s13157-010-0088-9.

[ece370383-bib-0017] Cushman, S. A. 2006. “Effects of Habitat Loss and Fragmentation on Amphibians: A Review and Prospectus.” Biological Conservation 128: 231–240.

[ece370383-bib-0018] Dewsbury, D. 2014. “A Novel, Effective and Safe Newt Trap.” Abhandlungen Aus Dem Westfälischen Museum Für Naturkunde 77: 189–208.

[ece370383-bib-0090] Dorazio, R. M. , and J. A. Royle . 2005. “Estimating Size and Composition of Biological Communities by Modeling the Occurrence of Species.” Journal of the American Statistical Association 100: 389–398.

[ece370383-bib-0019] Dorazio, R. M. , M. Kéry , J. A. Royle , and M. Plattner . 2010. “Models for Inference in Dynamic Metacommunity Systems.” Ecology 91: 2466–2475. 10.1890/09-1033.1. 20836468

[ece370383-bib-0020] Dorazio, R. M. , J. A. Royle , B. Söderström , and A. Glimskär . 2006. “Estimating Species Richness and Accumulation by Modeling Species Occurrence and Detectability.” Ecology 87: 842–854. 10.1890/0012-9658(2006)87[842:esraab]2.0.co;2. 16676528

[ece370383-bib-0021] Falaschi, M. , A. Melotto , R. Manenti , and G. F. Ficetola . 2020. “Invasive Species and Amphibian Conservation.” Herpetologica 76: 216–227. 10.1655/0018-0831-76.2.216.

[ece370383-bib-0022] Gelman, A. , X.‐L. Meng , and H. Stern . 1996. “Posterior Predictive Assessment of Model Fitness via Realized Discrepancies.” Statistica Sinica 6: 733–760.

[ece370383-bib-0023] Google Inc . 2023. https://www.google.com/earth/.

[ece370383-bib-0024] Grant, E. H. C. , D. A. Miller , B. R. Schmidt , et al. 2016. “Quantitative Evidence for the Effects of Multiple Drivers on Continental‐Scale Amphibian Declines.” Scientific Reports 6: 25625. 10.1038/srep25625. 27212145 PMC4876446

[ece370383-bib-0025] Guillera‐Arroita, G. 2017. “Modelling of Species Distributions, Range Dynamics and Communities Under Imperfect Detection: Advances, Challenges and Opportunities.” Ecography 40: 281–295. 10.1111/ecog.02445.

[ece370383-bib-0026] Guillera‐Arroita, G. , M. Kéry , and J. J. Lahoz‐Monfort . 2019. “Inferring Species Richness Using Multispecies Occupancy Modeling: Estimation Performance and Interpretation.” Ecology and Evolution 9: 780–792. 10.1002/ece3.4821. 30766668 PMC6362448

[ece370383-bib-0027] Guzy, J. C. , K. M. Halloran , J. A. Homyack , J. E. Thornton‐Frost , and J. D. Willson . 2019. “Differential Responses of Amphibian and Reptile Assemblages to Size of Riparian Buffers Within Managed Forests.” Ecological Applications 29: e01995. 10.1002/eap.1995. 31483894

[ece370383-bib-0028] Guzy, J. C. , S. J. Price , and M. E. Dorcas . 2014. “Using Multiple Methods to Assess Detection Probabilities of Riparian‐Zone Anurans: Implications for Monitoring.” Wildlife Research 41: 243–257. 10.1071/WR14038.

[ece370383-bib-0029] Halstead, B. J. , J. P. Rose , and P. M. Kleeman . 2021. “Time‐To‐Detection Occupancy Methods: Performance and Utility for Improving Efficiency of Surveys.” Ecological Applications 31: e2267. 10.1002/eap.2267. 33237597 PMC8047884

[ece370383-bib-0030] Hamer, A. J. , B. Barta , A. Bohus , B. Gál , and D. Schmera . 2021. “Roads Reduce Amphibian Abundance in Ponds Across a Fragmented Landscape.” Global Ecology and Conservation 28: e01663. 10.1016/j.gecco.2021.e01663.

[ece370383-bib-0031] Hamer, A. J. , and M. J. McDonnell . 2008. “Amphibian Ecology and Conservation in the Urbanising World: A Review.” Biological Conservation 141: 2432–2449. 10.1016/j.biocon.2008.07.020.

[ece370383-bib-0032] Hamer, A. J. , and K. M. Parris . 2013. “Predation Modifies Larval Amphibian Communities in Urban Wetlands.” Wetlands 33: 641–652. 10.1007/s13157-013-0420-2.

[ece370383-bib-0033] Hamer, A. J. , D. Schmera , and M. J. Mahony . 2021. “Multi‐Species Occupancy Modeling Provides Novel Insights Into Amphibian Metacommunity Structure and Wetland Restoration.” Ecological Applications 31: e2293. 10.1002/eap.2293. 33432692

[ece370383-bib-0034] Harka, A. , and Z. Sallai . 2004. Magyarország Halfaunája (Fish Fauna of Hungary). Szarvas, Hungary: Nimfea Termeszetvedelmi Egyesulet.

[ece370383-bib-0035] Hartel, T. , S. Nemes , D. Cogălniceanu , et al. 2007. “The Effect of Fish and Aquatic Habitat Complexity on Amphibians.” Hydrobiologia 583: 173–182. 10.1007/s10750-006-0490-8.

[ece370383-bib-0036] Holgerson, M. A. , A. Duarte , M. P. Hayes , et al. 2019. “Floodplains Provide Important Amphibian Habitat Despite Multiple Ecological Threats.” Ecosphere 10: e02853. 10.1002/ecs2.2853.

[ece370383-bib-0037] Houlahan, J. E. , C. S. Findlay , B. R. Schmidt , A. H. Meyer , and S. L. Kuzmin . 2000. “Quantitative Evidence for Global Amphibian Population Declines.” Nature 404: 752–755. 10783886 10.1038/35008052

[ece370383-bib-0038] Kéry, M. , R. M. Dorazio , L. Soldaat , A. Van Strien , A. Zuiderwijk , and J. A. Royle . 2009. “Trend Estimation in Populations With Imperfect Detection.” Journal of Applied Ecology 46: 1163–1172. 10.1111/j.1365-2664.2009.01724.x.

[ece370383-bib-0039] Kéry, M. , and J. A. Royle . 2008. “Hierarchical Bayes Estimation of Species Richness and Occupancy in Spatially Replicated Surveys.” Journal of Applied Ecology 45: 589–598. 10.1111/j.1365-2664.2007.01441.x.

[ece370383-bib-0040] Kloskowski, J. 2010. “Fish Farms as Amphibian Habitats: Factors Affecting Amphibian Species Richness and Community Structure at Carp Ponds in Poland.” Environmental Conservation 37: 187–194. 10.1017/S0376892910000494.

[ece370383-bib-0041] Kloskowski, J. , M. Nieoczym , and R. Stryjecki . 2020. “Between‐Habitat Distributions of Pond Tadpoles and Their Insect Predators in Response to Fish Presence.” Hydrobiologia 847: 1343–1356. 10.1007/s10750-020-04190-5.

[ece370383-bib-0042] Knapp, R. A. , and K. R. Matthews . 2000. “Non‐Native Fish Introductions and the Decline of the Mountain Yellow‐Legged Frog From Within Protected Areas.” Conservation Biology 14: 428–438.

[ece370383-bib-0043] Leibold, M. A. , and G. M. Mikkelson . 2002. “Coherence, Species Turnover, and Boundary Clumping: Elements of Meta‐Community Structure.” Oikos 97: 237–250. 10.1034/j.1600-0706.2002.970210.x.

[ece370383-bib-0044] Lima, N. G. S. , U. Oliveira , R. C. C. Souza , and P. C. Eterovick . 2019. “Dynamic and Diverse Amphibian Assemblages: Can We Differentiate Natural Processes From Human Induced Changes?” PLoS One 14: e0214316. 10.1371/journal.pone.0214316. 30913242 PMC6435182

[ece370383-bib-0045] Link, W. A. 1999. “Modeling Pattern in Collections of Parameters.” Journal of Wildlife Management 63: 1017–1027. 10.2307/3802817.

[ece370383-bib-0046] MacKenzie, D. I. , J. D. Nichols , G. B. Lachman , S. Droege , J. A. Royle , and C. A. Langtimm . 2002. “Estimating Site Occupancy Rates When Detection Probabilities Are Less Than One.” Ecology 83: 2248–2255.

[ece370383-bib-0047] MacKenzie, D. I. , J. D. Nichols , N. Sutton , K. Kawanishi , and L. L. Bailey . 2005. “Improving Inferences in Population Studies of Rare Species That Are Detected Imperfectly.” Ecology 86: 1101–1113.

[ece370383-bib-0048] MacKenzie, D. I. , and J. A. Royle . 2005. “Designing Occupancy Studies: General Advice and Allocating Survey Effort.” Journal of Applied Ecology 42: 1105–1114.

[ece370383-bib-0049] Mihaljevic, J. R. , M. B. Joseph , and P. T. J. Johnson . 2015. “Using Multispecies Occupancy Models to Improve the Characterization and Understanding of Metacommunity Structure.” Ecology 96: 1783–1792. 10.1890/14-1580.1. 26378301

[ece370383-bib-0050] Ministry of Agriculture . 2019. Development of an Ecosystem Base Map and Data Model. Budapest, Hungary: Ministry of Agriculture.

[ece370383-bib-0051] Monello, R. J. , and R. G. Wright . 2001. “Predation by Goldfish (*Carassius auratus*) on Eggs and Larvae of the Eastern Long‐Toed Salamander (*Ambystoma macrodactylum columbianum*).” Journal of Herpetology 35, no. 2: 350–353. 10.2307/1566132.

[ece370383-bib-0052] Nichols, J. D. , L. L. Bailey , A. F. O'Connell Jr. , et al. 2008. “Multi‐Scale Occupancy Estimation and Modelling Using Multiple Detection Methods.” Journal of Applied Ecology 45: 1321–1329. 10.1111/j.1365-2664.2008.01509.x.

[ece370383-bib-0053] Otto, C. R. V. , and G. J. Roloff . 2011. “Using Multiple Methods to Assess Detection Probabilities of Forest‐Floor Wildlife.” Journal of Wildlife Management 75: 423–431. 10.1002/jwmg.63.

[ece370383-bib-0054] Pacifici, K. , E. F. Zipkin , J. A. Collazo , J. I. Irizarry , and A. DeWan . 2014. “Guidelines for a Priori Grouping of Species in Hierarchical Community Models.” Ecology and Evolution 4: 877–888. 10.1002/ece3.976. 24772267 PMC3997306

[ece370383-bib-0055] Petitot, M. , N. Manceau , P. Geniez , and A. Besnard . 2014. “Optimizing Occupancy Surveys by Maximizing Detection Probability: Application to Amphibian Monitoring in the Mediterranean Region.” Ecology and Evolution 4: 3538–3549. 10.1002/ece3.1207. 25478146 PMC4224529

[ece370383-bib-0056] Phillott, A. D. , R. Speare , H. B. Hines , et al. 2010. “Minimising Exposure of Amphibians to Pathogens During Field Studies.” Diseases of Aquatic Organisms 92: 175–185. 10.3354/dao02162. 21268979

[ece370383-bib-0057] Plummer, M. 2017. “ JAGS Version 4.3.0 User Manual .” https://sourceforge.net/projects/mcmc‐jags/.

[ece370383-bib-0058] Préau, C. , P. Dubech , Y. Sellier , M. Cheylan , F. Castelnau , and D. Beaune . 2017. “Amphibian Response to the Non‐Native Fish, *Lepomis gibbosus*: The Case of the Pinail Nature Reserve, France.” Herpetological Conservation and Biology 12, no. 3: 616–623.

[ece370383-bib-0059] QGIS Development Team . 2022. “QGIS Geographic Information System.” Open Source Geospatial Foundation Project. http://qgis.osgeo.org.

[ece370383-bib-0060] R Core Team . 2023. R: A Language and Environment for Statistical Computing. Vienna, Austria: R Foundation for Statistical Computing. https://www.R‐project.org/.

[ece370383-bib-0061] Ribeiro, J. W., Jr. , T. Siqueira , G. L. Brejão , and E. F. Zipkin . 2018. “Effects of Agriculture and Topography on Tropical Amphibian Species and Communities.” Ecological Applications 28: 1554–1564. 10.1002/eap.1741. 29729054

[ece370383-bib-0062] Rowe, J. C. , A. Duarte , C. A. Pearl , et al. 2019. “Disentangling Effects of Invasive Species and Habitat While Accounting for Observer Error in a Long‐Term Amphibian Study.” Ecosphere 10: e02674. 10.1002/ecs2.2674.

[ece370383-bib-0063] Royle, J. A. , and J. D. Nichols . 2003. “Estimating Abundance From Repeated Presence‐Absence Data or Point Counts.” Ecology 84: 777–790.

[ece370383-bib-0064] Sankararaman, V. , S. Dalvi , D. A. W. Miller , and K. K. Karanth . 2021. “Local and Landscape Characteristics Shape Amphibian Communities Across Production Landscapes in the Western Ghats.” Ecological Solutions and Evidence 2: e12110. 10.1002/2688-8319.12110.

[ece370383-bib-0065] Schielzeth, H. 2010. “Simple Means to Improve the Interpretability of Regression Coefficients.” Methods in Ecology and Evolution 1: 103–113.

[ece370383-bib-0066] Schmidt, B. R. 2005. “Monitoring the Distribution of Pond‐Breeding Amphibians When Species Are Detected Imperfectly.” Aquatic Conservation: Marine and Freshwater Ecosystems 15: 681–692.

[ece370383-bib-0067] Schmidt, B. R. , R. I. Băncilă , T. Hartel , K. Grossenbacher , and M. Schaub . 2021. “Shifts in Amphibian Population Dynamics in Response to a Change in the Predator Community.” Ecosphere 12: e03528. 10.1002/ecs2.3528.

[ece370383-bib-0068] Schmidt, B. R. , S. S. Cruickshank , C. Bühler , and A. Bergamini . 2023. “Observers Are a Key Source of Detection Heterogeneity and Biased Occupancy Estimates in Species Monitoring.” Biological Conservation 283: 110102. 10.1016/j.biocon.2023.110102.

[ece370383-bib-0069] Sewell, D. , T. J. C. Beebee , and R. A. Griffiths . 2010. “Optimising Biodiversity Assessments by Volunteers: The Application of Occupancy Modelling to Large‐Scale Amphibian Surveys.” Biological Conservation 143: 2102–2110. 10.1016/j.biocon.2010.05.019.

[ece370383-bib-0070] Shaffer, H. B. , R. A. Alford , B. D. Woodward , S. J. Richards , R. G. Altig , and C. Gascon . 1994. “Quantitative Sampling of Amphibian Larvae.” In Measuring and Monitoring Biological Diversity. Standard Methods for Amphibians, edited by W. R. Heyer , M. A. Donnelly , R. W. McDiarmid , L. C. Hayek , and M. S. Foster , 130–141. Washington, DC: Smithsonian Institution Press.

[ece370383-bib-0071] Shulse, C. D. , R. D. Semlitsch , K. M. Trauth , and A. D. Williams . 2010. “Influences of Design and Landscape Placement Parameters on Amphibian Abundance in Constructed Wetlands.” Wetlands 30: 915–928. 10.1007/s13157-010-0069-z.

[ece370383-bib-0072] Snodgrass, J. W. , L. Bryan Jr. , and J. Burger . 2000. “Development of Expectations of Larval Amphibian Assemblage Structure in Southeastern Depression Wetlands.” Ecological Applications 10: 1219–1229.

[ece370383-bib-0073] Speybroeck, J. , W. Beukema , C. Dufresnes , et al. 2020. “Species List of the European Herpetofauna – 2020 Update by the Taxonomic Committee of the Societas Europaea Herpetologica.” Amphibia‐Reptilia 41: 139–189. 10.1163/15685381-bja10010.

[ece370383-bib-0074] Stolen, E. D. , D. M. Oddy , S. L. Gann , et al. 2019. “Accounting for Heterogeneity in False‐Positive Detection Rate in Southeastern Beach Mouse Habitat Occupancy Models.” Ecosphere 10: e02893. 10.1002/ecs2.2893.

[ece370383-bib-0075] Stuart, S. N. , J. S. Chanson , N. A. Cox , et al. 2004. “Status and Trends of Amphibian Declines and Extinctions Worldwide.” Science 306: 1783–1786. 15486254 10.1126/science.1103538

[ece370383-bib-0076] Su, Y. , and M. Yajima . 2021. “Package ‘R2jags’: Using R to Run ‘JAGS’.” Version 0.7‐1.

[ece370383-bib-0077] Tanadini, L. G. , and B. R. Schmidt . 2011. “Population Size Influences Amphibian Detection Probability: Implications for Biodiversity Monitoring Programs.” PLoS One 6: e28244. 10.1371/journal.pone.0028244. 22164250 PMC3229540

[ece370383-bib-0078] Teplitsky, C. , S. Plénet , and P. Joly . 2003. “Tadpoles' Responses to Risk of Fish Introduction.” Oecologia 134: 270–277. 10.1007/s00442-002-1106-2. 12647168

[ece370383-bib-0079] Thompson, W. L. 2004. Sampling Rare or Elusive Species: Concepts, Designs, and Techniques for Estimating Population Parameters. Washington, DC: Island Press.

[ece370383-bib-0080] Tóth‐Ronkay, M. , Z. Bajor , A. Bárány , et al. 2015. “Budapest.” In Vertebrates and Invertebrates of European Cities: Selected Non‐Avian Fauna, edited by J. G. Kelcey . New York, NY: Springer Science + Business Media.

[ece370383-bib-0081] Vörös, J. , I. Kiss , and M. Puky . 2015. “Conservation and Decline of Amphibians in Hungary.” In Amphibian Biology, Volume 11: Status of Conservation and Decline of Amphibians: Eastern Hemisphere, Part 4: Southern Europe and Turkey, edited by H. Heatwole and J. W. Wilkinson , 99–130. Exeter, UK: Pelagic Publishing.

[ece370383-bib-0082] Wassens, S. , A. Hall , and J. Spencer . 2017. “The Effect of Survey Method on the Detection Probabilities of Frogs and Tadpoles in Large Wetland Complexes.” Marine and Freshwater Research 68: 686–696. 10.1071/MF15183.

[ece370383-bib-0083] Weir, L. A. , J. A. Royle , P. Nanjappa , and R. E. Jung . 2005. “Modeling Anuran Detection and Site Occupancy on North American Amphibian Monitoring Program (NAAMP) Routes in Maryland.” Journal of Herpetology 39: 627–639.

[ece370383-bib-0084] Wellborn, G. A. , D. K. Skelly , and E. E. Werner . 1996. “Mechanisms Creating Community Structure Across a Freshwater Habitat Gradient.” Annual Review of Ecology and Systematics 27: 337–363.

[ece370383-bib-0085] Wells, K. D. 1977. “The Social Behaviour of Anuran Amphibians.” Animal Behaviour 25: 666–693.

[ece370383-bib-0086] Werner, E. E. , K. L. Yurewicz , D. K. Skelly , and R. A. Relyea . 2007. “Turnover in an Amphibian Metacommunity: The Role of Local and Regional Factors.” Oikos 116: 1713–1725.

[ece370383-bib-0087] Winandy, L. , E. Darnet , and M. Denoël . 2015. “Amphibians Forgo Aquatic Life in Response to Alien Fish Introduction.” Animal Behaviour 109: 209–216. 10.1016/j.anbehav.2015.08.018.

[ece370383-bib-0088] Winandy, L. , and M. Denoël . 2013. “Introduced Goldfish Affect Amphibians Through Inhibition of Sexual Behaviour in Risky Habitats: An Experimental Approach.” PLoS One 8: e82736. 10.1371/journal.pone.0082736. 24312432 PMC3843724

[ece370383-bib-0089] Zipkin, E. F. , A. DeWan , and J. Andrew Royle . 2009. “Impacts of Forest Fragmentation on Species Richness: A Hierarchical Approach to Community Modelling.” Journal of Applied Ecology 46: 815–822. 10.1111/j.1365-2664.2009.01664.x.

