# Supplementary material for: Improving Inference Within Freshwater Community Studies: Accounting for Variable Detection Rates of Amphibians and Fish
Source: Ecol Evol. 2024 Oct 16;14(10):e70383. doi: 10.1002/ece3.70383 (PMC11483536; doi:10.1002/ece3.70383)
Supplement: Supplementary file 1 — Table S1 Table S2 Table S3 [file ECE3-14-e70383-s001.pdf]

SUPPORTING INFORMATION

Improving inference within freshwater community studies: accounting for variable detection rates of amphibians and fish

Ecology and Evolution

Andrew J. Hamer[1,2], Júlia Horányi[1,2]

[1] Institute of Aquatic Ecology, HUN-REN Centre for Ecological Research, Karolina út. 29, 1113 Budapest, Hungary

[2] National Multidisciplinary Laboratory for Climate Change, HUN-REN Centre for Ecological Research, Karolina út. 29, 1113 Budapest, Hungary

*Corresponding author:

Andrew J. Hamer, Institute of Aquatic Ecology, HUN-REN Centre for Ecological Research, Karolina út. 29, 1113 Budapest, Hungary

Email: hamer.andrew.j@gmail.com

ORCID ID: https://orcid.org/0000-0001-6031-7841

Table S1. Pearson correlation coefficients (*r*) among three covariates describing survey effort during three surveys.

	VES_1	VES_2	VES_3	Sweeps_1	Sweeps_2	Sweeps_3	Traps_1	Traps_2	Traps_3
VES_1	-	0.807	0.683	0.806	0.804	0.728	0.622	0.589	0.532
VES_2	0.807	-	0.760	0.773	0.822	0.652	0.603	0.652	0.528
VES_3	0.683	0.760	-	0.761	0.760	0.826	0.538	0.516	0.656
Sweeps_1	0.806	0.773	0.761	-	0.954	0.898	0.512	0.517	0.540
Sweeps_2	0.804	0.822	0.760	0.954	-	0.872	0.535	0.555	0.523
Sweeps_3	0.728	0.652	0.826	0.898	0.872	-	0.483	0.469	0.613
Traps_1	0.622	0.603	0.538	0.512	0.535	0.483	-	0.954	0.823
Traps_2	0.589	0.652	0.516	0.517	0.555	0.469	0.954	-	0.811
Traps_3	0.532	0.528	0.656	0.540	0.523	0.613	0.823	0.811	-

VES = number of minutes conducting call/ visual encounter surveys; Sweeps = number of dip-net sweeps; Traps = number of newt traps deployed at a pond.

Table S2. Summary of hyper-parameters for occupancy (α) and detection (β) covariates of amphibian communities for: (1) call/ visual encounter surveys (VES, 100 ponds surveyed); (2) dip-netting (98 ponds); (3) newt trapping (41 ponds). Estimates include 95% Bayesian credible intervals (2.5^{th} and 97.5^{th} percentiles of the posterior distribution). Strongly influential relationships for beta estimates of the covariates are where ≥ 0.9 of the posterior weight of the mean estimate is above or below zero (highlighted in bold, except intercept coefficients). Bayesian *p*-values for Models 2 – 4 indicated acceptable model fit (*p* = 0.455, 0.462 and 0.389, respectively).

μ = beta estimate (mean community response); σ = standard deviation in the response to the covariate across species; SD = standard deviation

Community-level hyper-parameter		Mean	SD	2.5^{th}	97.5^{th}
Model 2	VES				
$\mu_{\alpha 0}$	Intercept	–0.682	0.464	–1.514	0.316
$\sigma_{\alpha 0}$	Intercept	1.497	0.581	0.738	2.993
$\mu_{\beta 0}$	Intercept	–0.497	0.561	–1.571	0.645
$\sigma_{\beta 0}$	Intercept	2.452	1.018	0.835	4.633
$\mu_{\beta 1}$	Days	–0.306	0.431	–1.177	0.565
$\sigma_{\beta 1}$	Days	1.436	0.818	0.380	3.583
$\boldsymbol{\mu_{\beta 2}}$	**Days^2**	**–0.819**	**0.453**	**–1.667**	**0.174**
$\sigma_{\beta 2}$	Days^2	1.069	0.859	0.045	3.322
$\boldsymbol{\mu_{\beta 3}}$	**Effort**	**1.039**	**0.428**	**0.126**	**1.848**

Community-level hyper-parameter		Mean	SD	2.5th	97.5th
$\sigma_{\beta 3}$	Effort	0.799	0.750	0.031	2.904
Model 3	Dip-netting				
$\mu_{\alpha 0}$	Intercept	–0.682	0.464	–1.514	0.316
$\sigma_{\alpha 0}$	Intercept	1.497	0.581	0.738	2.993
$\mu_{\beta 0}$	Intercept	–0.497	0.561	–1.571	0.645
$\sigma_{\beta 0}$	Intercept	2.452	1.018	0.835	4.633
$\mu_{\beta 1}$	Days	–0.306	0.431	–1.177	0.565
$\sigma_{\beta 1}$	Days	1.436	0.818	0.380	3.583
$\boldsymbol{\mu_{\beta 2}}$	**Days^2**	**–0.819**	**0.453**	**–1.667**	**0.174**
$\sigma_{\beta 2}$	Days^2	1.069	0.859	0.045	3.322
$\boldsymbol{\mu_{\beta 3}}$	**Effort**	**1.039**	**0.428**	**0.126**	**1.848**
$\sigma_{\beta 3}$	Effort	0.799	0.750	0.031	2.904
Model 4	Newt traps				
$\mu_{\alpha 0}$	Intercept	–0.553	0.627	–1.669	0.761
$\sigma_{\alpha 0}$	Intercept	1.920	1.043	0.296	4.400
$\mu_{\beta 0}$	Intercept	–0.754	0.652	–1.997	0.553
$\sigma_{\beta 0}$	Intercept	2.498	1.383	0.157	4.853
$\mu_{\beta 1}$	Days	–0.258	0.571	–1.360	0.901
$\sigma_{\beta 1}$	Days	2.415	1.274	0.252	4.800
$\mu_{\beta 2}$	Days^2	–0.500	0.617	–1.686	0.730
$\sigma_{\beta 2}$	Days^2	3.154	1.109	0.990	4.902

Community-level hyper-parameter		Mean	SD	2.5th	97.5th
$\mu_{\beta 3}$	Effort	0.094	0.538	–0.991	1.144
$\sigma_{\beta 3}$	Effort	1.105	0.992	0.035	3.828

Days = number of days since 19 March 2023; Days^2 = quadratic effect of Days; Effort = survey effort (VES: number of minutes conducting call/ visual encounter surveys; Dip-netting: number of dip-net sweeps; Newt traps: number of newt traps deployed).

Table S3. Summary of species-specific estimates for occupancy (α) and detection (β) covariates for nine amphibian species detected using three separate survey methods (call/ visual encounter surveys [VES], dip-netting and newt trapping). Estimates include 95% Bayesian credible intervals (2.5^{th} and 97.5^{th} percentiles of the posterior distribution). Strongly influential relationships for beta estimates of the covariates are where ≥ 0.9 of the posterior weight of the mean estimate is above or below zero (highlighted in bold, except intercept coefficients).

SD = standard deviation.

Species	Species-specific parameter		Mean	SD	2.5^{th}	97.5^{th}
Model 2: VES						
Bombina bombina	α_0	Intercept	–0.604	0.787	–1.749	1.225
	β_0	Intercept	–3.396	1.539	–6.778	–0.861
	β_1	Days	–0.423	0.642	–1.812	0.774
	β_2	$Days^2$	–0.854	0.647	–2.232	0.405
	$\boldsymbol{\beta_3}$	**Effort**	**1.422**	**0.628**	**0.441**	**2.908**
Bufo bufo	α_0	Intercept	–1.482	0.551	–2.462	–0.293
	β_0	Intercept	–1.785	1.520	–5.104	1.012
	β_1	Days	0.016	0.731	–1.462	1.521

Species	Species-specific parameter		Mean	SD	2.5th	97.5th
	**β_2**	**Days^2**	**–1.105**	**0.721**	**–2.765**	**0.158**
	**β_3**	**Effort**	**1.532**	**0.690**	**0.496**	**3.191**
Bufotes viridis	α_0	Intercept	–2.389	0.439	–3.289	–1.554
	β_0	Intercept	0.702	1.491	–2.071	3.930
	β_1	Days	–0.992	0.974	–3.236	0.642
	**β_2**	**Days^2**	**–1.288**	**0.922**	**–3.531**	**0.249**
	**β_3**	**Effort**	**1.744**	**1.360**	**0.100**	**5.547**
Hyla arborea	α_0	Intercept	–0.669	0.608	–1.601	0.665
	β_0	Intercept	–2.265	1.295	–5.129	–0.064
	β_1	Days	–0.243	0.585	–1.470	0.905
	**β_2**	**Days^2**	**–1.206**	**0.663**	**–2.761**	**–0.113**
	**β_3**	**Effort**	**1.428**	**0.594**	**0.506**	**2.827**
Lissotriton vulgaris	α_0	Intercept	–2.303	0.475	–3.246	–1.360
	β_0	Intercept	0.026	1.433	–2.810	3.002
	β_1	Days	0.567	0.963	–1.123	2.749

Species	Species-specific parameter		Mean	SD	2.5th	97.5th
	**β_2**	**Days^2**	**–1.305**	**0.925**	**–3.538**	**0.239**
	β_3	Effort	0.597	0.703	–0.987	1.796
Pelobates fuscus	α_0	Intercept	–0.884	0.812	–2.069	1.044
	β_0	Intercept	–2.652	1.579	–6.052	0.137
	**β_1**	**Days**	**–1.213**	**0.979**	**–3.579**	**0.267**
	**β_2**	**Days^2**	**–2.006**	**1.148**	**–4.869**	**–0.527**
	**β_3**	**Effort**	**1.160**	**0.591**	**0.073**	**2.449**
Pelophylax spp. complex	α_0	Intercept	0.939	0.425	0.264	1.911
	β_0	Intercept	1.216	0.795	–0.081	3.024
	**β_1**	**Days**	**0.571**	**0.374**	**–0.070**	**1.401**
	**β_2**	**Days^2**	**–1.359**	**0.545**	**–2.595**	**–0.508**
	**β_3**	**Effort**	**1.536**	**0.563**	**0.654**	**2.812**
Rana dalmatina	α_0	Intercept	–1.181	0.528	–2.025	0.018
	β_0	Intercept	–2.194	1.375	–5.227	0.177
	**β_1**	**Days**	**–2.417**	**1.467**	**–6.177**	**–0.509**

Species	Species-specific parameter		Mean	SD	2.5th	97.5th
	β_2	Days^2	0.142	1.178	–1.476	3.135
	β_3	Effort	0.916	0.614	–0.428	2.069
Model 3: Dip-netting						
Bombina bombina	α_0	Intercept	–2.196	1.275	–4.217	0.791
	β_0	Intercept	–4.364	2.950	–10.492	0.890
	β_1	Days	1.068	1.455	–1.627	4.247
	β_2	Days^2	–0.973	1.733	–4.834	2.141
	β_3	Effort	0.687	0.724	–1.025	1.952
Bufo bufo	α_0	Intercept	–1.305	0.423	–2.051	–0.394
	β_0	Intercept	0.232	1.594	–2.824	3.541
	β_1	Days	–0.990	0.968	–3.209	0.613
	**$\boldsymbol{\beta_2}$**	**Days^2**	**–2.466**	**1.132**	**–5.041**	**–0.631**
	β_3	Effort	0.798	0.555	–0.362	1.935
Bufotes viridis	α_0	Intercept	–2.850	0.499	–3.906	–1.947
	β_0	Intercept	2.797	2.319	–1.323	7.825

Species	Species-specific parameter		Mean	SD	2.5th	97.5th
	β_1	Days	–0.291	1.205	–2.838	2.012
	**β_2**	**Days^2**	**–2.155**	**1.496**	**–5.434**	**0.505**
	β_3	Effort	0.669	0.785	–1.251	2.015
Hyla arborea	α_0	Intercept	–1.282	0.665	–2.312	0.215
	β_0	Intercept	–0.920	1.886	–4.678	2.873
	β_1	Days	0.956	1.024	–0.877	3.202
	**β_2**	**Days^2**	**–3.335**	**1.402**	**–6.480**	**–1.054**
	**β_3**	**Effort**	**0.875**	**0.551**	**–0.181**	**2.087**
Lissotriton vulgaris	α_0	Intercept	–0.650	0.252	–1.137	–0.150
	β_0	Intercept	0.333	0.882	–1.379	2.135
	β_1	Days	0.484	0.556	–0.571	1.640
	β_2	Days^2	–0.041	0.678	–1.378	1.333
	β_3	Effort	0.672	0.478	–0.360	1.575
Pelobates fuscus	α_0	Intercept	–0.851	0.417	–1.562	0.042
	β_0	Intercept	0.414	1.464	–2.364	3.480

Species	Species-specific parameter		Mean	SD	2.5th	97.5th
	β_1	Days	0.790	0.739	–0.577	2.364
	**β_2**	**Days^2**	**–3.082**	**1.080**	**–5.435**	**–1.259**
	**β_3**	**Effort**	**1.053**	**0.577**	**0.129**	**2.445**
Pelophylax spp. complex	α_0	Intercept	0.488	0.840	–0.607	2.612
	β_0	Intercept	–4.583	1.371	–7.353	–2.088
	**β_1**	**Days**	**1.912**	**0.665**	**0.786**	**3.354**
	β_2	Days^2	0.623	0.677	–0.651	2.041
	**β_3**	**Effort**	**0.956**	**0.450**	**0.178**	**1.976**
Rana dalmatina	α_0	Intercept	–0.777	0.314	–1.351	–0.111
	β_0	Intercept	1.832	1.388	–0.678	4.791
	**β_1**	**Days**	**–1.953**	**1.000**	**–4.196**	**–0.288**
	**β_2**	**Days^2**	**–4.122**	**1.306**	**–6.937**	**–1.907**
	**β_3**	**Effort**	**0.843**	**0.509**	**–0.144**	**1.941**
Triturus dobrogicus	α_0	Intercept	–2.642	0.782	–4.049	–0.977
	β_0	Intercept	–1.164	2.544	–6.444	3.734

Species	Species-specific parameter		Mean	SD	2.5th	97.5th
	**β_1**	**Days**	**2.018**	**1.702**	**–0.572**	**6.151**
	β_2	Days^2	–1.514	1.624	–4.930	1.615
	**β_3**	**Effort**	**1.000**	**0.730**	**–0.199**	**2.804**
Model 4: Newt traps						
Bufo bufo	α_0	Intercept	–0.393	1.587	–2.396	3.911
	β_0	Intercept	–1.624	2.006	–5.780	2.617
	β_1	Days	–0.126	1.660	–3.507	3.366
	**β_2**	**Days^2**	**–4.955**	**2.569**	**–10.963**	**–1.103**
	β_3	Effort	0.458	1.231	–1.669	3.468
Bufotes viridis	α_0	Intercept	–2.025	1.645	–4.786	1.742
	β_0	Intercept	–2.577	2.634	–8.609	2.056
	β_1	Days	–1.853	2.301	–7.572	1.648
	β_2	Days^2	–1.809	2.395	–6.986	2.793
	β_3	Effort	–0.136	1.563	–3.853	2.782
Lissotriton vulgaris	α_0	Intercept	0.005	1.393	–1.514	3.933

Species	Species-specific parameter		Mean	SD	2.5th	97.5th
	β_0	Intercept	–2.857	1.665	–6.714	–0.309
	**β_1**	**Days**	**–1.518**	**1.005**	**–3.932**	**–0.009**
	β_2	Days^2	0.183	0.989	–1.768	2.280
	β_3	Effort	–0.164	0.916	–2.265	1.448
Pelobates fuscus	α_0	Intercept	–1.257	1.574	–3.530	2.787
	β_0	Intercept	–2.435	2.206	–7.416	1.490
	β_1	Days	–0.589	1.637	–4.193	2.584
	**β_2**	**Days^2**	**–2.391**	**2.102**	**–7.190**	**1.259**
	β_3	Effort	0.252	1.262	–2.197	3.109
Pelophylax spp. complex	α_0	Intercept	–1.543	1.490	–3.708	2.259
	β_0	Intercept	–3.364	2.666	–9.496	0.741
	**β_1**	**Days**	**2.707**	**2.474**	**–0.733**	**8.740**
	β_2	Days^2	–0.235	1.929	–4.128	3.774
	β_3	Effort	0.161	1.039	–2.039	2.307
Rana dalmatina	α_0	Intercept	0.530	1.628	–1.404	4.926

Species	Species-specific parameter		Mean	SD	2.5th	97.5th
	β_0	Intercept	–1.259	1.553	–4.432	2.047
	β_1	Days	–0.840	1.372	–3.976	1.645
	**β_2**	**Days^2**	**–4.777**	**2.189**	**–9.869**	**–1.464**
	β_3	Effort	0.413	1.076	–1.488	2.983
Triturus dobrogicus	α_0	Intercept	–2.323	0.946	–4.079	–0.307
	β_0	Intercept	–1.267	2.103	–5.894	2.939
	**β_1**	**Days**	**–2.655**	**2.372**	**–8.546**	**0.585**
	β_2	Days^2	0.041	2.024	–3.847	4.396
	β_3	Effort	–0.089	1.629	–3.885	3.066

See Table S2 for a description of the parameters.
